# Quantitative trait loci and genes associated with salmonid alphavirus load in Atlantic salmon: implications for pancreas disease resistance and tolerance

**DOI:** 10.1038/s41598-020-67405-8

**Published:** 2020-06-25

**Authors:** M. L. Aslam, D. Robledo, A. Krasnov, H. K. Moghadam, B. Hillestad, R. D. Houston, M. Baranski, S. Boison, N. A. Robinson

**Affiliations:** 1 Breeding and Genetics, Nofima (Norwegian Institute of Food Fisheries and Aquaculture Research), Postboks 210, 1431 Ås, Norway; 2 The Roslin Institute and Royal (Dick) School of Veterinary Studies, University of Edinburgh, Edinburgh, UK; 3 Benchmark Genetics Norway AS, Sandviksboder 3A, Bergen, Norway; 4 Mowi Genetics AS, Sandviksboder 77AB, Bergen, Norway; 5 Sustainable Aquaculture Laboratory-Temperate and Tropical (SALTT), School of BioSciences, The University of Melbourne, Parkville, 3010 Australia

**Keywords:** Transcriptomics, Genetic markers

## Abstract

Salmonid alphavirus infection results in pancreas disease causing severe economic losses for Atlantic salmon aquaculture. Knowledge about genes and pathways contributing to resistance is limited. A 54 K SNP panel was used to genotype 10 full-sibling families each consisting of ~ 110 offspring challenged with salmonid alphavirus subtype 3. Relative heart viral load was assessed at 4- and 10-weeks post-infection using quantitative PCR. A moderate genomic heritability of viral load at 4 weeks (0.15–0.21) and a high positive correlation with survival (0.91–0.98) were detected. Positions of QTL detected on chromosome 3 matched those for survival detected by other studies. The SNP of highest significance occurred in the 3′ untranslated region of *gig1*, a fish-specific antiviral effector. Locus B of immunoglobulin heavy chain mapped to an area containing multiple SNPs with genome-wide association. Heart mRNA-seq comparing parr from families with high- versus low-genomic breeding value, and matching sample genotypes for SNPs, identified two eQTL for salmonid alphavirus load. Immune genes associated with trans-eQTL were numerous and spread throughout the genome. QTL regions contained several genes with known or predicted immune functions, some differentially expressed. The putative functional genes and variants identified could help improve marker-based selection for pancreas disease resistance.

## Introduction

Infectious diseases are a major threat to the sustainability of Atlantic salmon farming, causing production and economic losses, and having a negative impact on fish welfare and the environment. One of the major disease problems in Atlantic salmon aquaculture is pancreas disease (PD), which affects mainly first year Atlantic salmon smolts^1–8^. Mortality due to natural outbreaks of PD is variable, ranging from negligible up to 63%^7,9–12^. A Norwegian cohort study (2006–2008) reported an average PD-associated mortality of ~ 6.9% (range 0.7–26.9%)^13^. However, economic losses due to morbidity can be high due to prolonged loss of appetite, growth retardation, and reduced filet quality^7,11^. There have been several reports describing after-effects of PD outbreaks including the development of runts, and significantly lower growth in PD affected fish^11,14–16^.

PD is caused by *Salmonid alphavirus* (SAV), an infectious single-stranded positive-sense RNA virus^17,18^. The mode of the spread of infection of salmonid diseases between co-habitants in water, and the ability to perform controlled disease challenge tests on large families of Atlantic salmon, provides us with a convenient model to use for investigating genes affecting resistance to this alphavirus, and the knowledge generated using such models might provide leads for the treatment or prevention of alphavirus disease in other species.

Globally, six subtypes of SAV virus have been identified^19^, each distributed over a specific geographic range^8,20^. Two subtypes cause outbreaks of PD in Norway: SAV2, mainly affecting farms in the north, and SAV3, mainly in mid and south Norway, with no overlap or co-infection within sites^3,21^. Regardless of SAV subtype, internal symptoms of the disease include yellow mucoid gut contents or empty intestines, faecal casts, circulatory disturbance and petechial haemorrhages in the periacinar fat^22^. Histological investigation often reveals complete loss of exocrine pancreatic tissue, cardiac myocytic necrosis and inflammation and degeneration and/or inflammation of skeletal muscle^23^. Real-time reverse transcription-quantitative PCR (RT-qPCR) can be used to diagnose PD, identify SAV subtypes and measure relative SAV viral load among individuals^24^.

Salmon farmers apply different measures to prevent and control PD outbreaks including vaccination. However, vaccinations tested using different SAV subtypes show substantial variation in efficacy, suggesting a complex, multifactorial basis for defence^12,22,25^. The spread of PD mainly occurs through horizontal transmission^8,26,27^, and vertical transmission has not been convincingly demonstrated^28,29^. Hence, restrictions have been applied in Norway for the movement of infected fish to avoid spread of PD. However, it is almost impossible to prevent transmission of SAV via water currents. In this context, genetic improvement of host resistance through selective breeding is a feasible alternative to reduce the impact of the virus on Atlantic salmon production. Resistance to PD in Atlantic salmon has been shown to be moderate to highly heritable with estimates ranging from 0.21 to 0.54 depending on the population used and the model of analysis applied^30,31^. Selection methods employing molecular tools, i.e. marker assisted selection (MAS) and genomic selection (GS), have been shown to have higher accuracy, and result in higher genetic gains than conventional phenotypic selection for traits which cannot be directly measured on selection candidates^32^, like PD disease resistance. MAS can be more efficient than GS when major QTL explaining a large proportion of the genetic variance for the trait have been identified. Gonen et al.^31^ reported a QTL for resistance to SAV3 in Atlantic salmon (survival data from both fresh and seawater controlled challenge tests and low density SNP genotypes) which showed a strong signal (explaining ~ 23% of within family variance for the trait) and consistency across two populations. This QTL for survival against SAV3, located on chromosome Ssa03, has been confirmed in Norwegian Mowi AS and Benchmark Genetics Norway AS populations (Baranski M, Gonen S, Norris A, Hjelmeland R, Sonesson A, Boison S in prep. and Hillestad B, Makvandi-Nejad S, Krasnov A, Meuwissen T and Moghadam H, in prep. respectively) using intraperitoneal challenge of large numbers of nucleus families (150 to 220) genotyped using a 55 K SNP array (personal communication).

Knowledge of the biology of resistance to PD have been revealed by comparing the transcriptome of fish with high- and low-genomic breeding values for PD survival at time points before and after challenge with SAV3^33^. Naïve salmon with high genomic breeding values for resistance show a higher expression of genes involved in early antiviral responses compared to naïve salmon with relatively low genomic breeding values for PD resistance. Four weeks post-infection, genes involved in the acquired immune response are more highly expressed in salmon with high-compared to low-genomic breeding values for PD resistance. Earlier mobilization of acquired immunity could accelerate clearance of the pathogen and resolution of the disease.

Viral load and histopathological damage to heart tissue after infection with SAV3 is lower in fish with high- than fish with low-genomic breeding values for PD resistance^33^. Lower early viral replication has been found to be associated with resistance to viral disease in salmonid species e.g. rainbow trout resistance to infectious hematopoietic necrosis virus^34^. Viral load might be a more economically valuable, accurate and convenient measure of resistance or tolerance to PD in the field because mortalities in the field due to PD are often negligible (even though hosts carry the virus and shed into the environment) and because animals that are better able to resist and clear the SAV infection will be under less stress and more likely to perform better (in terms of growth and survival).

In the current study, our two primary aims were to further explore the underlying genetics affecting PD resistance in Atlantic salmon and to examine the potential of using SAV3 viral load as an alternative phenotype. The experiments in this study therefore explore the genetic basis of resistance against PD using two phenotypes, (a) survival (dead/alive) and (b) relative SAV3 viral load measured using RT-qPCR based cycle threshold (*C*_*t*_) values. The specific objectives of the study were to, (i) estimate and compare genetic parameters (heritability, genetic correlations) for both traits; (ii) map QTL for SAV viral load and compare to the published QTL for survival; (iii) map eQTL in genomic regions significantly associated with resistance to PD; (iv) identify genes with potential immune function mapping to the QTL regions that could have functions affecting PD resistance.

## Results

### Challenge test trials

The distribution of mortalities across high and low breeding value (EBV) families was described in detail in Robinson et al.^33^. Twelve percent mortality was observed over the duration of both trials with higher mortality in the fish with low genomic estimated breeding value (L-gEBV) than high genomic breeding value (H-gEBV) as expected (16.9 and 4.3% mortality in the L- and H-gEBV IP challenge and 20.2 and 7.1% mortality in the L- and H-gEBV CH challenge groups respectively). Samples for *C*_*t*_ were obtained at 4 weeks post-infection (W4), which coincided with the highest daily mortality for the cohabitant challenge and after the IP challenge had completed the highest mortality period. Samples were also obtained at 10 weeks post-infection (W10). W4 IP animals had a lower viral load than CH animals (Table 1), which was expected considering that IP tested fish survived the steepest part of the mortality curve. The IP trial would therefore be expected to be less powerful for detecting QTL as viral titre was generally lower in IP than CH challenged fish, and as the most susceptible fish in the L-gEBV group would have died prior to viral load measurement (and therefore are not in the analysis). IP fish also presented lower viral loads at W10 by which stage the virus would have been substantially cleared in some animals (Table 1). Viral loads were lower at W10 than at W4 for both challenge models which is likely to be because the immune system has responded and substantially cleared the virus by W10 and because the most susceptible animals would have succumbed to the disease and died by W10 (Table 1). No mortalities were observed in the control group of uninfected fish.

**Table 1:** Summary of SAV RT-qPCR cycle threshold (C_t_) results.

Sampling time	Challenge type	EBV group	n	Mean	SD	Minimum	Maximum
W4	CH	H	141	17.99	2.01	11.96	24.48
W4	CH	L	125	17.47	1.66	12.58	23.20
W4	IP	H	140	23.28	2.08	17.02	30.16
W4	IP	L	116	22.65	2.18	17.32	30.32
W10	CH	H	122	27.50	2.02	22.47	38.09
W10	CH	L	85	26.53	1.51	22.80	33.21
W10	IP	H	131	29.98	3.15	24.63	39.23
W10	IP	L	107	29.99	3.52	18.68	39.89

C_t_ is negatively related to relative viral load. Salmon with high or low estimated genomic breeding values (H or L gEBV group respectively) were infected for the challenge test trials by intraperitoneal injection (IP) or cohabitation with infected fish (CH). Testing occurred 4 weeks post-infection (W4) and 10 weeks post-infection (W10).

### Genomics based variance components

Overall, genomic heritability for resistance to SAV3 ranged from 0.15 to 0.74 depending on challenge type and trait (Table 2). Higher genomic heritability was detected for the CH trial and for the dead or alive survival trait (*P*_*DA*_) than for the IP trial and *C*_*t*_ trait respectively. Genetic correlations computed between ${P}_{DA}$ and $Ct$ using the bivariate model ranged from $0.91 (SE \,\, 0.28)$ to $0.98 \left(SE \,\, 0.05\right)$ for IP and CH respectively and also varied with data type ($Ct$ and ${P}_{DA}$ traits analyzed separately for CH and IP or all together). The genetic correlation for $Ct$ values between IP and CH was 0.73 (SE 0.20, Table 3). The genetic correlation of $Ct$ values between sampling times (W4 and W10) was moderate for CH (0.58 ± 0.35) and high for the IP challenge model (0.99 ± 0.26, Table 3).

**Table 2:** Heritability estimates for survival (dead or alive, ${\mathrm{P}}_{\mathrm{D}\mathrm{A}}$) and relative SAV3 viral load (SAV3 RT-qPCR cycle threshold, $\mathrm{C}\mathrm{t}$) phenotypes at W4.

Traits	${h}^{2}$	
	IP	CH
${P}_{DA}$	0.40 (0.13)	0.74 (0.08)
*C*_*t*_	0.15 (0.06)	0.21 (0.07)

Salmon were infected for the challenge test trials by intraperitoneal injection (IP) or cohabitation with infected fish (CH). Standard errors in parentheses.

**Table 3:** Genetic correlations for relative SAV3 viral load (SAV3 RT-qPCR cycle threshold, C_t_) between sampling times (W4 and W10) and across and within challenge types.

Comparison	Correlation (standard error)
W4 vs. W10 (CH)	0.58 (0.35)
W4 vs. W10 (IP)	0.99 (0.26)
CH vs. IP	0.73 (0.20)

Standard errors in parentheses.

*IP* intraperitoneal injection, *CH* cohabitation with infected fish.

### Genome wide association analysis

**Figure 1:**
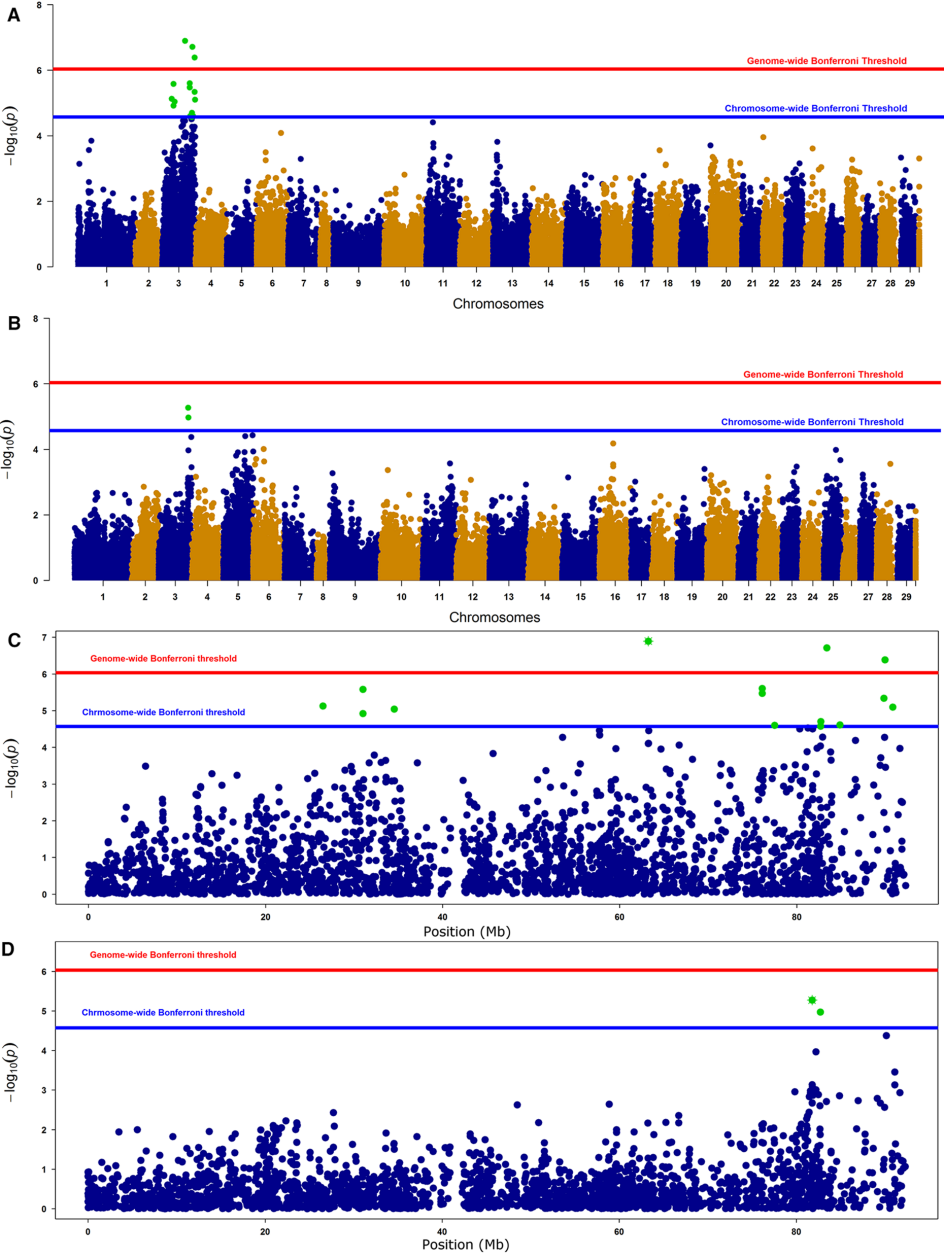
Manhattan plots of GWAS p-values for relative SAV3 viral load (− C_t_) distributed across all chromosomes and for positions across Ssa03. Markers crossing genome and/or chromosome wide Bonferroni threshold are dotted in green colour. Results from the CH challenge trial (**A**, **C**) and the IP trial (**B**, **D**) are shown for SNPs on all chromosomes (**A**, **B**) and for SNPs on Ssa03 (**C**, **D**).

GWAS analysis of the salmonid alpha virus RT-qPCR $Ct$-value trait detected likely genome-wide QTL positioned on Ssa03. Two SNPs showed significant association (chromosome-wide significance) with relative SAV viral load for the analysis of W4 individuals in the IP test (Table 4, Fig. 1C), and 15 SNPs around the same region had chromosome- or genome-wide Bonferroni corrected significance for the analysis of individuals in the CH test (Table 4, Fig. 1A). No evidence for multiple QTL was found when the top-most significant marker was fitted as a covariant in the analysis, even though the SNPs of genome-wide significance mapped to a broad region of Ssa03. The estimated size of the QTL effect (percentage of the genetic variation explained) for the top most significant SNPs at position 63 Mb (CH) and 83 Mb (IP) was 34.7 and 48.6% respectively. The λ value (magnitude of the deviation inflation/deflation of *p*-values) for the analysis was 1.21 (distribution of *p*-values presented as a Q–Q plot in Supplemental Fig. S1).

**Table 4:** SNPs with genome- and chromosome-wide significant associations with relative SAV viral load in CH and IP trials.

CHR	SNP	Pos (bp)	A1	A2	MAF	$\alpha$	SE	*P*-value
**CH**								
3	AX-87986229	26488333	A	C	0.34	− 0.74	0.17	7.43E−06
3	AX-87865538	30994059	A	G	0.34	− 0.72	0.16	1.2E−05
3	AX-96308943	31004919	C	T	0.37	− 0.74	0.16	2.63E−06
3	AX-87618835	34527144	T	C	0.27	− 0.74	0.17	9.06E−06
3	AX-87849339	63236952	G	A	0.49	0.83	0.16	1.28E−07
3	AX-96264043	76082530	C	A	0.30	− 0.73	0.16	3.36E−06
3	AX-96455227	76082537	C	A	0.30	− 0.74	0.16	2.48E−06
3	AX-88146078	77489246	A	G	0.22	− 0.78	0.19	2.51E−05
3	AX-88163075	8678000	T	C	0.17	− 0.88	0.21	2.66E−05
3	AX-98305314	82718110	G	T	0.30	− 0.68	0.16	1.99E−05
3	AX-88293328	83391111	T	C	0.49	0.85	0.16	1.94E−07
3	AX-88010358	84875109	C	T	0.36	− 0.63	0.15	2.44E−05
3	AX-96235336	89833309	A	G	0.47	0.69	0.15	4.58E−06
3	AX-88234566	89963083	C	T	0.48	0.83	0.16	4.1E−07
3	AX-88122450	90830375	C	G	0.39	0.58	0.13	7.96E−06
**IP**								
3	AX-87885147	81776059	A	C	0.21	− 1.20	0.26	5.33E−06
3	AX-88130071	82718000	C	T	0.19	− 1.22	0.28	1.07E−05

*Pos (bp)* physical position of SNP in base pairs, *A1 & A2* minor & major alleles, respectively, *MAF* minor allele frequency, *α* Allele substitution effect, *SE* standard error, *P* significance value.

The map position for the QTL for relative SAV viral load detected on Ssa03 was unclear but the area containing SNPs significantly associated with *C*_*t*_ covered the same positions as for genome- and chromosome-wide associations detected for *P*_*DA*_ (eight SNPs positioned between 75–83 Mb and three between 89–91 Mb) and the position detected by Gonen et al.^31^ for survival to IP challenge, strongly suggesting that both are caused by the same underlying gene(s). This conclusion is supported by the high correlation of *C*_*t*_ and *P*_*DA*_ traits found in this study (close to 1.0).

### IGH and B cell-specific genes

**Figure 2:**
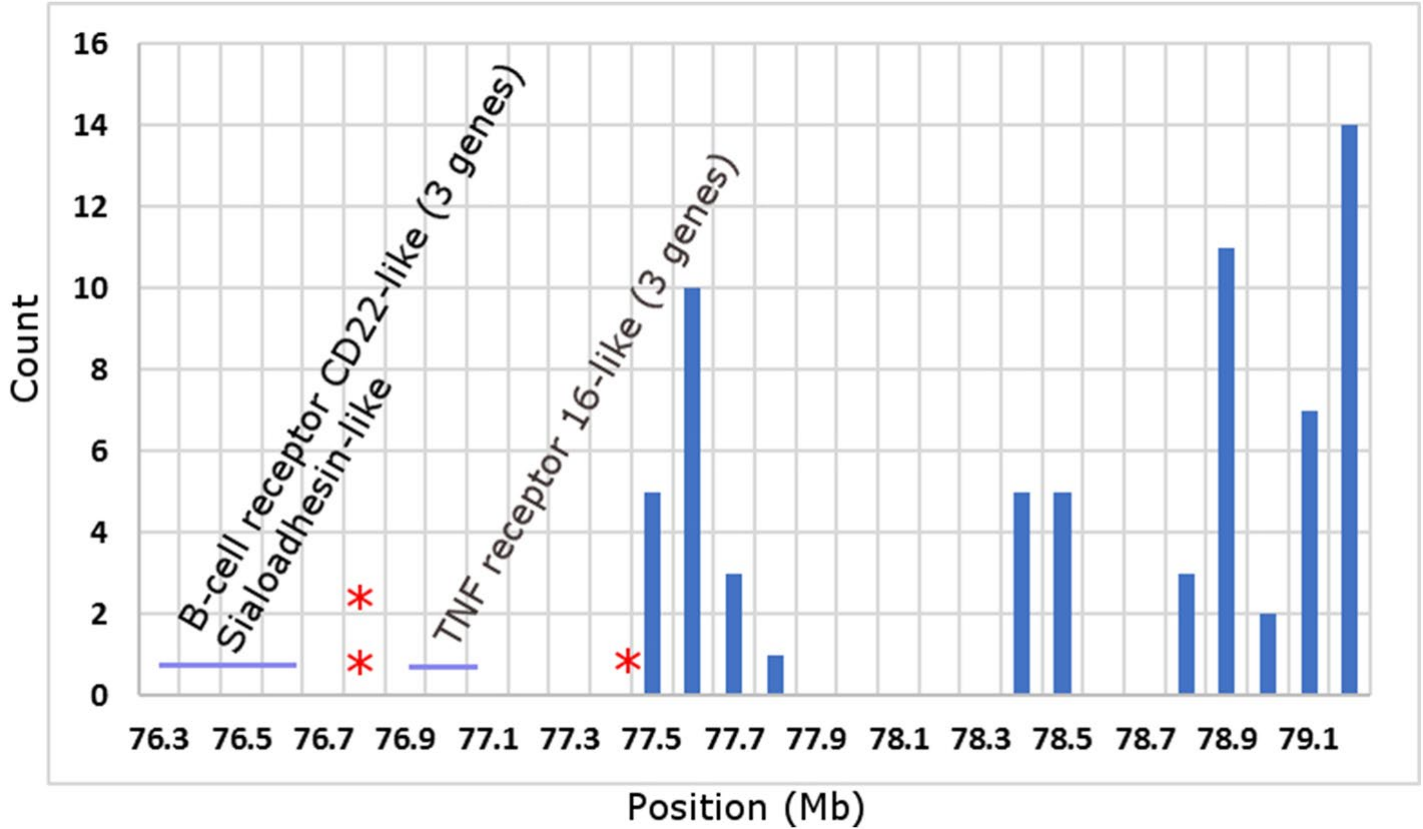
Histograms showing counts of sequences of IGH locus mapping across Ssa03 (positions in Mega base pairs, Mbp). Location of immune genes and SNPs significantly associated with C_t_ (red asterisks) are indicated.

The region containing SNPs associated with the SAV viral load includes locus B of Atlantic salmon immunoglobulin heavy chain (IGH). Reference sequences of the constant regions of all three types of immunoglobulins (IgM, IgT and IgD) and multiple VH, D and J segments of the variable region retrieved from The International Immunogenetics Information System^35^ (https://www.imgt.org/), 106 sequences in total, map between 77.5 and 79,3 Mb (Fig. 2). A short 258-Kb region between 76.4–76.5 contains four genes with important roles in B cells. *cd22* has been found to regulate B-cell receptors in mammals preventing excessive activity and autoimmune reactions^36^. Strong responses of multiple *cd22* paralogs to vaccination have been shown in Atlantic salmon^37^. Three other genes located in the same area belong to the TNF receptor family that controls all aspects of B-cell development and function^38^. Genes mapping between 76.3–77.0 Mb are similar to TNF receptor type 16, which is also known as *ngfr*—receptor of nerve growth factor. Human NGFR was reported to rescue B lymphocytes from apoptosis^39^, but the precise role of these genes in salmon is unknown. Overall, a 2.8 Mb region on Ssa03 contains 87 genes in addition to the IGH segments (Fig. 2). Four genes from the B-cell signalling pathway (*ras-related c3 botulinum toxin substrate 1, gtpase hras, grb2-related adapter protein* and *serine/threonine-protein kinase smg1*) map between 80 and 92 Mb.

### Characterization of SNPs and genes mapping to the distal end of Ssa03

We undertook a more detailed investigation of the variants and genes mapping to a region between 88 and 93 Mb towards the end of Ssa03 which included the three most distally occurring chromosome and genome-wide associated *C*_*t*_ QTL SNP loci, and encompassed the most likely QTL position estimated by Gonen et al.^31^ and a likely QTL position estimated from yet unpublished studies of the Mowi AS and Benchmark Genetics Norway AS populations (Baranski M, Gonen S, Norris A, Hjelmeland R, Sonesson A, Boison S in prep. and Hillestad B, Makvandi-Nejad S, Krasnov A, Meuwissen T and Moghadam H, in prep. respectively) for PD survival (88–93 Mb toward the end of Ssa03, Fig. 1C).

**Figure 3:**
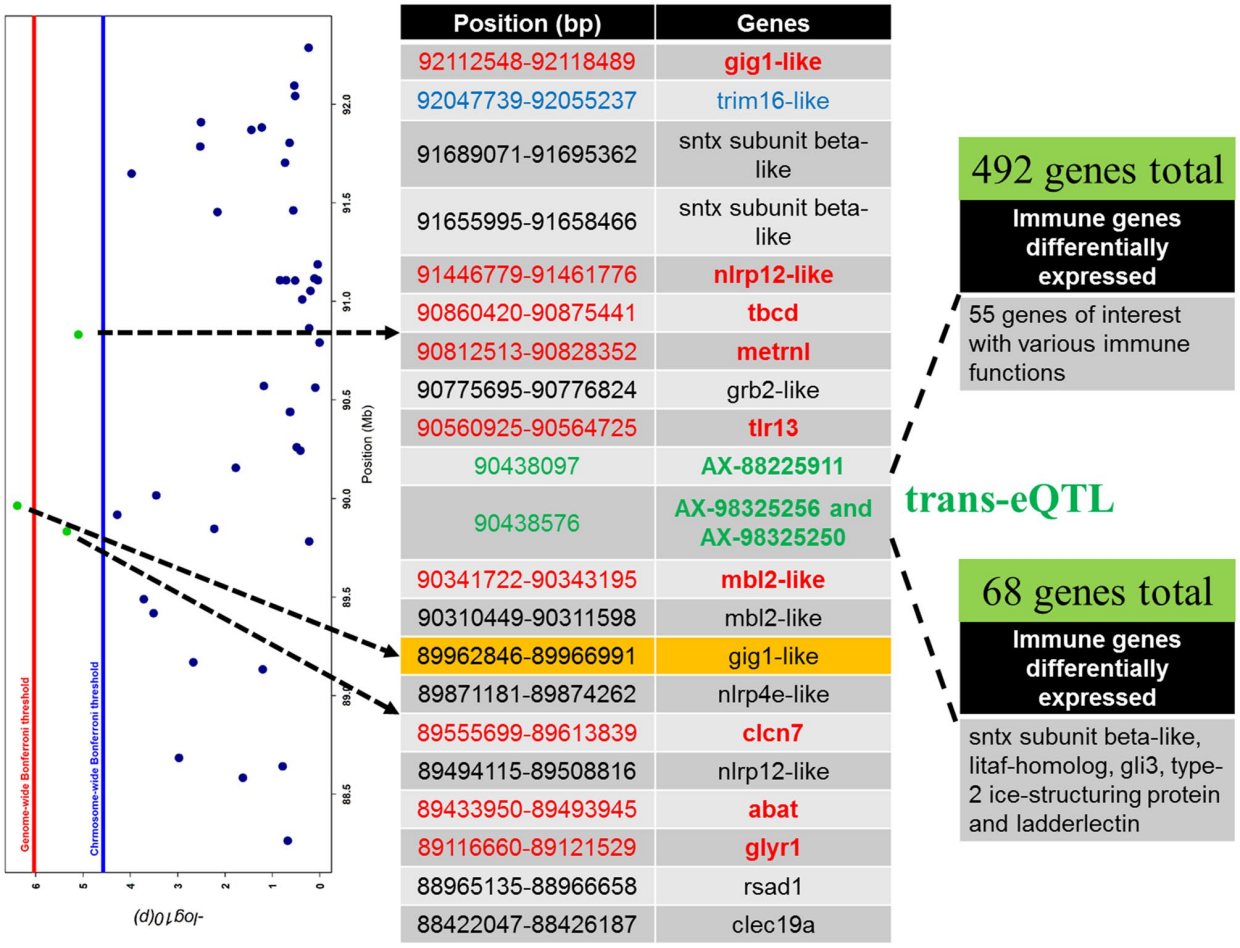
Map of the 88–93 Mb QTL region on Ssa03 showing Manhattan plot and genes that are differentially expressed in H-gEBV versus L-gEBV salmon (highlighted as red text) and trans-eQTL (highlighted in green text). SNP AX-88234566 at position 89963083 which was associated with relative SAV3 viral load (genome wide significance) mapped to a 3′UTR region of gig1 (highlighted in orange). The other two SNPs associated with relative SAV3 viral load mapped to intergenic regions (relative positions indicated by arrows).

**Figure 4:**
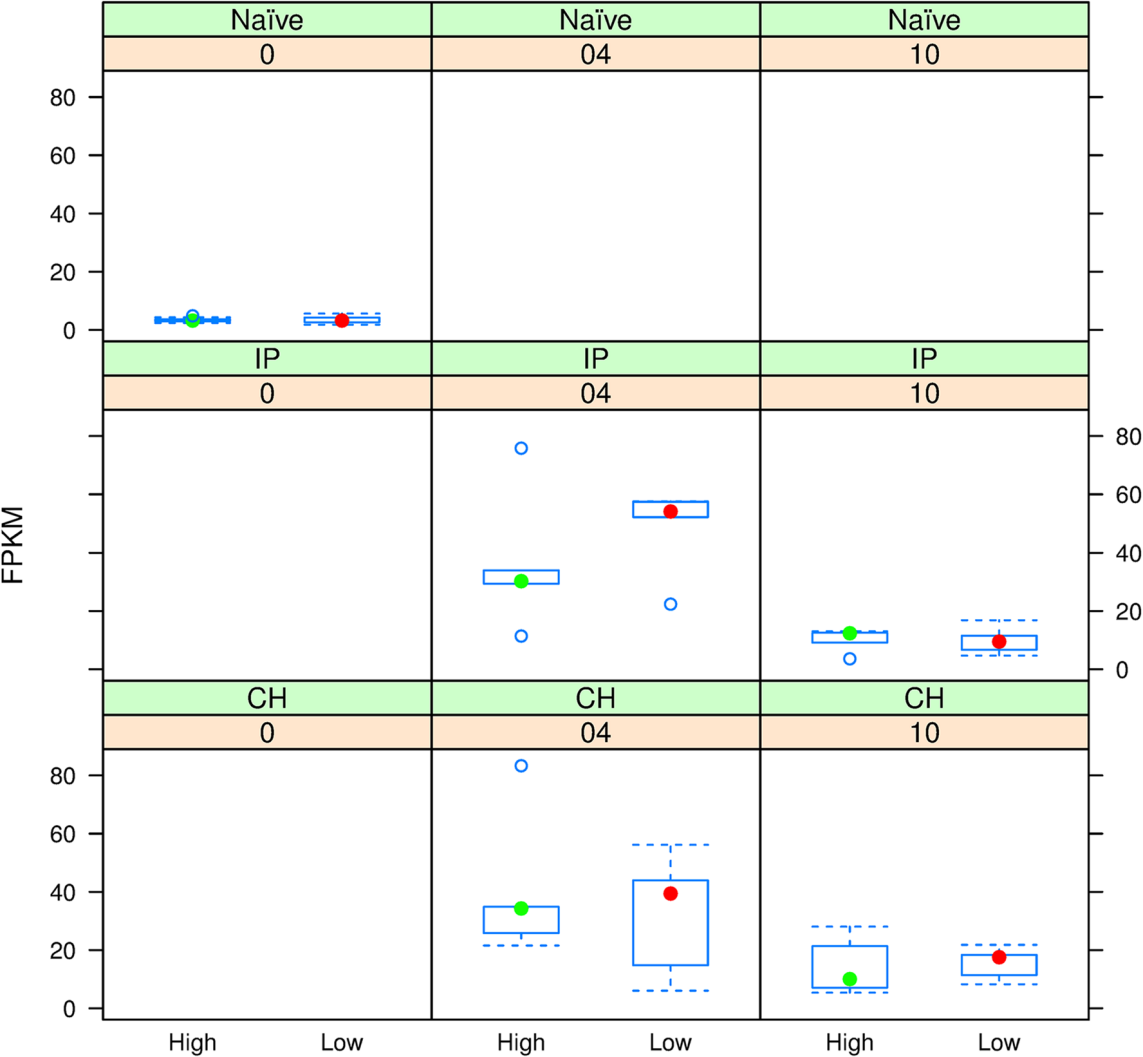
Boxplot of changes in expression of grass carp reovirus-induced gene 1 (Gig1-like), mapping to approximately 90.0 Mb along Ssa03. Plots show count of fragments per kilobase million (FPKM) for H-gEBV (green dot) and L-gEBV (red dot) fish challenged by intraperitoneal injection (IP) or cohabitation (CH) and sampled at 0- (naïve), 4- or 10-weeks post-infection.

Ninety-eight genes mapped to this 88–93 Mb region on Ssa03. Several of these genes are known or are predicted to have immune functions (Table 5, Fig. 3). The SNP with genome-wide association with *C*_*t*_ in this region (AX-88234566 at position 89963083) maps to the 3′ untranslated region of grass carp reovirus-induced gene 1 (*gig1*)*,* a member of a multigene family of antiviral effectors found only in fish^40^. *Rsad1* or *viperin* destroys lipid rafts preventing budding and release of viruses^41^. Atlantic salmon *gig1* and *viperin* strongly respond to viral infections in salmon^42,43^. *Tlr13* is a putative ortholog of mammalian pathogen recognition receptor binding bacterial RNA^44^. Proteins with NACHT, LRR and PYD domains can be involved in diverse processes including pathogen recognition and inflammation^45,46^. *Mbl12* may activate the lectin complement pathway. Several genes mapping to this region of Ssa03 were differentially expressed between H- and L-gEBV fish at stages before and/or after infection. *Tlr13* was up-regulated in H-gEBV relative to L-gEBV in naïve fish and at 4 weeks post infection, and down-regulated in H-gEBV relative to L-gEBV fish 10 weeks post infection. *Gig1*-like was significantly down-regulated in expression in H-gEBV compared to L-gEBV fish at 4 weeks post-IP-infection (Fig. 4). The regulation of expression of *gig1* was positively correlated with viral load over all treatment groups and over all weeks (Pearson’s r^2^ = 0.73, *P* < 0.01). This reflects the differences in expression and viral load over the weeks as no significant correlations were found within W0, W4 or W10 (both viral load and level of gene expression starts low at W0, becomes relatively higher at W4 and falls back to low levels by W10). Table 5 also shows differentially expressed genes with metabolic and developmental roles.

**Table 5:** Highlighted immune genes mapping to 88–93 Mb QTL region on Ssa03.

Gene name	Position
C-type lectin domain family 19, member A (*clec19a*)	88422047–88426187
Radical S-adenosyl methionine domain-containing protein 1, mitochondrial-like (*rsad1*)	88965135–88966658
NACHT, LRR and PYD domains (*nlrp12*-like)	89494115–89508816
NACHT, LRR and PYD domains (*nlrp4e*-like)	89871181–89874262
Grass Carp Reovirus-Induced gene 1 (*Gig1*-like)*	89962846–89966991
Mannose-binding protein C-like (*mbl2*-like)*	90310449–90311598
Toll-like receptor 13 (*tlr13*)*	90560925–90564725
NACHT, LRR and PYD domains (*nlrp12*-like)*	91446779–91461776
NACHT, LRR and PYD domains (sntx subunit beta)	91655995–91658466
NACHT, LRR and PYD domains (sntx subunit beta)	91689071–91695362
Putative oxidoreductase GLYR1 (*glyr1*)*	89116660–89121529
Chloride channel-2C voltage-sensitive 7 (*clcn7*)*	89555699–89613839
Meteorin, glial cell differentiation regulator*	90812513–90828352
Tubulin-specific chaperone D (*tbcd*)*	90860420–90875441
4-aminobutyrate aminotransferase (*abat*)*	89433950–89493945

Start and end map positions for each gene are shown (bp). Differentially expressed genes are marked with *.

Eighty-three missense mutations, 1 lost stop codon in putative oxidoreductase (*glyr1*) and 1 premature stop codon in tripartite motif-containing protein 16 (*trim16*) were detected. Missense mutations were detected in several genes of interest, i.e. two in genes affecting pathogen recognition and inflammation (*nlrp4* and *nlrp12*), 1 toll-like receptor (*tlr13*), 1 ubiquitin peptidase that interacts with herpes virus (*usp7*), 2 lectins (c-type lectin , *clec19a*, and mannose-binding protein C, *mbl2*, the latter of which activates the lectin complement pathway), 3 genes involved in methylation (*mettl22*, *suz12* and *glyr1*) and 1 gene inhibiting apoptosis through casp9 (*arl6ip1*).

### eQTL

Two potential eQTL of interest were detected on Ssa03, one which was marked by SNP AX-88225911 at position 89781579 and associated with 492 genes (*p*-value < 0.01), and another eQTL which was marked by two SNPs (AX-98325256 at position 904438097 and AX-98325250 at position 90438576) and associated with 68 genes (Fig. 3 and Supplemental Tables S1, S2, and S3). The former (AX-88225911) showed the highest association with an ubiquitin-like protein and a probable E3 ubiquitin-protein ligase RNF144A-A; several other ubiquitin-related genes showed nominal association (E3 ubiquitin-protein ligase SMURF2, E3 ubiquitin-protein ligase AMFR, or ubiquitin-conjugating enzyme E2 variant 1), and other immune genes such as major histocompatibility complex class I-related gene, interleukin-4 receptor alpha chain, tumor necrosis factor receptor superfamily member 1B or C–C motif chemokine 19. The latter eQTL (AX-98325256 and AX-98325250) showed FDR-corrected significant association with the stonustoxin subunit beta-like gene (*sntx* beta-like). A gene also annotated as *sntx* beta-like mapped to around 91.6 Mb along Ssa03 less than 1 Mb from SNP AX-88122450 which was significantly associated with relative SAV viral load (Supplemental Tables S1, S3, Fig. 3). *Sntx* beta-like was more highly expressed in H-gEBV than L-g-EBV fish 4 weeks post-infection in the CH trial.

## Discussion

### Clearance and resistance to initial infection

This study is the first comparison of viral load and survival genetic parameters for a fish species. Mortality due to PD is often negligible, but the disease severely affects growth, feed conversion and meat quality^47^. Animals able to restrict viral load are likely to be less stressed, grow faster and produce a higher quality product. Furthermore, infected salmon that survive but carry high viral loads probably have a greater influence on the spread and impact of disease outbreaks than other survivors with lower relative SAV viral load. Relative SAV viral load (*C*_*t*_) measures the ability to resist the initial SAV infection and clear the virus and holds promise as a more relevant trait for genomic selection to improve resistance to pancreas disease than the ability to survive infection as measured by *P*_*DA*_. Despite *C*_*t*_ having a lower heritability than *P*_*DA*_, the two traits were found to be highly correlated, and the same QTL region at the proximal end of Ssa03 was found to be associated with both traits. Together with our previous reported finding of lower viral load in H-gEBV fish^33^, this confirms expectations that survival is at least partly dependent on the ability to resist and clear the virus. Tests of viral load therefore have potential application for assessing the relative resistance of fish even in the absence of significant mortality caused by pancreas disease. Animals able to restrict viral replication are likely to be less stressed, grow faster and produce a higher quality product.

Fish with PD survive due to either clearance of the virus or ability to tolerate the pathogen. Measurement of viral load enables discrimination of survivors that could become carriers and spread infection^48–50^. Breeding for low viral load would select individuals that mount an active defense to clear the virus and would therefore be a more attractive strategy than breeding for survival. Mapping of *Ct* and *P*_*DA*_ QTL to the same position suggests that defence is of paramount importance. The difference between the CH and IP models is likely to reflect the greater complexity of the processes involved in the CH trial, since water borne pathogen encounters barrier tissue (skin, gill and intestine) and mucosal immunity^44^. Slower and more natural development of the disease with the CH challenge would also provide greater scope for mounting acquired immune responses. This may explain the detection of more SNPs significantly associated with *C*_*t*_ on Ssa03, and the suggestion of additional QTL peaks on Ssa03 for the CH trial compared to the IP trial. However, these differences can also be at least partly accounted for by the design of experiment. Sampling for the measurement of *C*_*t*_ at 4 weeks post infection occurred as the CH trial underwent and the IP trial finished the steepest phase of the mortality curve. In this way the SAV RT-qPCR *C*_*t*_ measurements gave a snapshot of relative SAV viral load at different phases of infection in the CH and IP trial animals. We would expect that the greater difference between salmon with high and low resistance at the peak of disease could also lead to the greater sensitivity of the CH compared to IP trials for detecting *C*_*t*_ QTL.

Gonen et al.^31^ found evidence that similar genetic mechanisms affect resistance to PD in fry and post-smolt, post-smolt being the stage usually affected by natural outbreaks of PD. Therefore, it seems likely that the QTL associated with resistance and clearance of the virus in freshwater will also be responsible for resistance and clearance of the pathogen in saltwater at later life stages. *P*_*DA*_ heritability for the IP challenge test in our study was greater than that found by Norris et al.^30^ for mortality data recorded after an outbreak of the disease in the sea, but similar to that found by Gonen et al.^31^ in post-smolt salmon that were experimentally challenged by IP injection (using similar methodology to that presented here).

### Candidate genes of immune function mapping to QTL

Twelve chromosome- or genome-wide viral load associated SNPs mapped to the distal end of Ssa03 from 76 Mb onwards. This broad region containing SNPs associated with PD resistance corresponds with the findings of the unpublished studies by Mowi AS and Benchmark Genetics Norway AS (Baranski M, Gonen S, Norris A, Hjelmeland R, Sonesson A, Boison S in prep. and Hillestad B, Makvandi-Nejad S, Krasnov A, Meuwissen T and Moghadam H, in prep. respectively), which were able to differentiate the presence of two QTL, QTL1 mapping to 88–93 Mb, and QTL2 mapping to 73–78 Mb. Given the pivotal role of active defence for resistance against PD, the immune genes in this area are of special interest. The region of QTL2 begins with several B cell-specific genes and the IGH locus. Studies of transcriptome responses of Atlantic salmon to viral infections have suggested that acquired humoral immunity is of key importance for resistance against viral diseases. Increased expression of *ig* in the heart and spleen was identified as the main factor explaining longer survival in fish challenged with Infectious Salmon Anaemia—ISA^51^. Recovery from Heart and Skeletal Muscle Inflammation (HSMI) and PD occurs shortly after marked increase of *ig* transcripts in the infected sites^43^. The association of the immunoglobulin locus with the viral load QTL is supported by our findings of higher expression of *ig light chain* in H-gEBV than L-gEBV fish and our finding of higher activity of several genes associated with the recruitment of B-cells nuclear gtpase slip-gc^52^, cd22^36^, in H-gEBV fish at 4 weeks post-SAV infection^33^. The genomic regions encoding IGH were identified as duplicated loci by Yakuike et al.^53^ and IGH maps to this region of Ssa03 and to an area of Ssa06 in the Atlantic salmon genome^54^. Publications in preparation (Hillestad B, Makvandi-Nejad S, Krasnov A, Meuwissen T and Moghadam H, in prep.) show that the 10 most genome-wide significant SNPs in the QTL2 region (*P* < 4.0E−20) map to 73–78 Mb which encompasses at least part of the same region containing IGH-B, which, according to our estimates, produces about 70% of all antibodies^55^. IGH-B includes multiple exons encoding the constant regions of IgM, IgT and IgD, 28 D (diversity) genes, eight JH (junction) genes and multiple VH (variable) genes of which 36 are functional^56^. In each B cell, the IGH locus encodes a single heavy chain produced by somatic recombination of four genes (CH, JH, D and V) followed with diversification—enzymatic addition and deletion of nucleotides. Mutations in the IGH-B locus may affect the amounts and properties of antibodies (recognition of pathogens and affinity) and the composition of the antibody repertoire.

The clearest positional information for the QTL1 was for the Mowi unpublished study (which had the largest set of families and SNPs, Baranski M, Gonen S, Norris A, Hjelmeland R, Sonesson A, Boison S in prep.) where the *P*_*DA*_ QTL1 mapped to between 88 and 93 Mb towards the end of Ssa03. Both the *C*_*t*_ and *P*_*DA*_ QTL are likely to be caused by the same underlying gene(s) given the high genetic correlation (nearly 1.0) detected between the traits in our study. Therefore, we also focussed our search for functionally relevant candidate immune genes and eQTL to the region where QTL1 was positioned in this unpublished study.

The region between 88 and 93 Mb towards the end of Ssa03 contained genes, that bind to foreign organisms and activate the complement pathway, such as pattern recognition c-type lectins *mbl2*-like which binds mannose, fucose and N-acetylglucosamine (e.g. binds to mannose glycans on HIV^57^), activates the lectin complement pathway and enhances engulfment by phagocytes^58,59^, and *clec19a* which adheres to pathogen infected cells and is involved in apoptosis^60^. Other genes mapping in the region (or affected by SNPs in the region) included *tlr13* which is expressed in macrophages and recognises conserved molecules expressed by viruses^61^ and other microbes, *sntx* subunit beta-like which may be a member of the membrane attack complex destroying infected cells^62^, *grb2*-like involved in T-cell specific protein tyrosine kinase and mannose receptor signalling^63,64^, *nlrp12*-like could be a negative regulator of the innate immune response^65^ and plays a role in mediating viral inflammatory responses^66^ and *gig1*-like which is an antiviral effector^40^.

### QTL and nature of genetic resistance to PD

The QTL detected account for a large proportion of the genetic variance for the C_t_ trait. However, the estimation of effect size is likely to be upwardly biased in our experiment because high- and low-ranking families for PD resistance were studied, which in effect resulted in a form of selective genotyping for GWAS. Most genetic studies assume that QTL of large effect (in the extreme case, when a single locus is responsible for nearly all of the genetic variation in the trait) are caused by the inheritance of two, or a few, alternative alleles of a single gene (e.g. one or a few causative single nucleotide polymorphisms or indels). However, the hypervariable and multi-element nature of the IGH locus presents us with a quite different scenario, one which might explain why the strength of association and positioning of QTL2 on Ssa03 has been found to vary depending on population and challenge test event studied.

Antibodies are assembled using different combinations of these hypervariable elements. These antibodies recognise viral antigens and set the immune response in process. The hypervariability provided by multiple combinations of elements, and diversification with enzymatic addition and deletion of nucleotides, is needed because infectious organisms like SAV quickly mutate and change, and the immune system of vertebrates needs to be able to present antibodies that can recognise new strains and forms of pathogens as they arise. The IGH-B locus is characterized by its large size and extreme complexity, and several immune genes, including genes with key roles in B cells, map to the same region as IGH-B. The variants in the multiple elements of IGH-B may have additive effects on the resistance afforded by the locus.

This rapidly evolving and complex interaction between the virus and the IGH locus might explain why the SNPs in genome-wide association with relative viral load (*C*_*t*_) map broadly to positions from 63 to 90 Mb across Ssa03 in our and the other studies and why the strength of association for the second QTL peak at 73–78 Mb varies depending on the salmon population studied and challenge test occasion (presumably using different viral homogenates or isolates)^31^ (and unpublished data from Mowi AS and Benchmark Genetics Norway AS : Baranski M, Gonen S, Norris A, Hjelmeland R, Sonesson A, Boison S in prep. and Hillestad B, Makvandi-Nejad S, Krasnov A, Meuwissen T and Moghadam H in prep. respectively).

Breeding to select entire haplotypes with preferable combinations of IGH-B variants could achieve rapid genetic improvement in resistance in the short-term. But breeding to promote diversity at the locus should also be considered because selection to fix variation at one element would also fix variation at closely mapping elements in LD. By utilising gene editing it might be possible to create specific IGH-B elements that give the individual high resistance to current strains of the PD virus while maintaining diversity among other nearby mapping elements. In the future it could become feasible to sequence prevalent strains of virus and predict what IGH-B variants would be most effective and should be created using gene editing as a gene therapy.

On-the-other-hand, the position of the other more pronounced and consistent QTL1 peak close to the end of Ssa03 could indicate the involvement of one or a few genes and a simpler underlying biological explanation for the effect on resistance at this position. Further studies are needed to determine if and to what extent these two QTL interact to effect resistance, precisely what variants are involved and how breeding strategies should be formulated. The hypervariable multi-element nature of the IGH locus might necessitate the adoption of novel approaches for these further investigations.

## Conclusion

Heart salmonid alphavirus load at 4 weeks post infection is heritable and highly correlated with survival (dead or alive) and is therefore a good indicator of pancreas disease resistance in Atlantic salmon. Viral load after cohabitant challenge tests accounts for a broader range of defence mechanisms, and is more sensitive for the detection of QTL, than viral load after intraperitoneal challenge. The QTL detected on Ssa03 for the viral load trait are likely the same as those detected for survival. The two QTL explained a high proportion of the total genetic variance for the trait. A suite of immune genes with potential effects on resistance map in the vicinity of the QTL including the multi-gene IGH-B locus*, cd22-like,* c-type lectins *mbl2*-like and *clec19a*, *tlr13*, *sntx* subunit beta-like, *grb2*-like, *nlrp12*-like and *gig1*-like, and SNPs acting as trans-eQTL on genes of other chromosomes including *sntx* subunit beta-like. It is possible that the causative variant(s) on Ssa03 affects the expression of multiple immune genes in *cis*- and *trans*-association. The study represents the most comprehensive search for candidate genes mapping to the region of the salmonid alphavirus load/pancreas disease resistance QTL to date and has contributed useful insights about the biology of resistance to viral diseases in salmon.

## Methods

### Resource population

A population comprising 10 full-sib families (~ 110 fish per family) originating from the breeding nucleus of Benchmark Genetics Norway AS was used for the current study (as described by^33^). Out of these 10 families, 5 were produced from parents which ranked high and 5 were from parents that ranked low for resistance (measured as the probability of being dead or alive, P_DA_, with an intraperitoneal challenge test against SAV3 with point deviation from mean genomic breeding value, gEBV, of + 27.3% and − 14.3% for the high- and low- families respectively).

### Challenge test

Atlantic salmon were challenged with SAV3 using two models, cohabitation (CH) infection and intraperitoneal (IP) injection as described previously^33^. Parr stage fish (average weight of ~ 30 g) tagged with passive integrated transponders (PIT-tags, parentage recorded) were split into two groups by randomly sampling ~ 50 individuals per family. Parr in the replicate tanks were acclimatized for ~ 20 days before being challenged. Half the parr in each tank were anaesthetized and challenged by intraperitoneal injection with 0.1 ml of SAV3 suspension (isolate VESO Vikan Jnr. 2,333 at 2.5 × 10^7^ virus/ml). PIT tags of these fish were scanned, and these animals recorded as the (IP group). The remaining fish from each tank were anesthetised, PIT tag recorded and transferred to the same tank as the IP group fish. These fish were recorded as cohabitants with the IP infected fish (CH group fish) and the IP infected fish in this way acted as shedders of the SAV virus for the cohabitant challenge. The environmental conditions including feeding, nutrition, water temperature and salinity were therefore uniform across the different challenge tests (common garden experiment). Mortalities, PIT tag number and date were recorded daily. The total duration of the challenge experiment was 10 weeks. Adipose fin clips were sampled after 4 weeks post-challenge from all animals and preserved in 85% ethanol for DNA extraction for SNP genotyping.

All challenge testing procedures were performed by VESO Vikan in a secure containment facility in Namsos Norway using standard operating procedures as approved by the Norwegian Animal Research Authority, National Assignments Department (approval no. 8841). Both challenges were conducted in accordance with the laws and regulations controlling experiments and procedures for live animals in Norway (the Animal Welfare Act of December 20th, 1974, No 73, chapter VI sections 20–22 and the Regulation on Animal Experimentation of January 15th, 1996).

### DNA extraction and genotyping

Genomic DNA was extracted from the fin clips using a commercial kit (DNeasy Blood & Tissue Kit, Qiagen), following the manufacturer’s instructions. All individuals (n ~ 1,100) were genotyped using a ~ 55 K axiom Affymetrix SNP Genotyping Array (NOFSAL03).

Genotypic data was filtered using the Plink software^67^ excluding SNPs with minor allele frequency (MAF) lower than 5%, missing more than 15% of genotypes, and/or with one or more Mendelian inheritance error. Approximately 54 K SNPs were retained for downstream analyses.

### Sampling for RT-qPCR

Samples were collected at three different time points: (i) the day before challenge with SAV3 at which stage fish were naïve to the virus (designated week 0 of the challenge trial, W0), (ii) at week 4 (W4) and (iii) at week 10 (W10) of the SAV3 challenge trial. Fish from each family and challenge model were randomly chosen and sacrificed at these time points to acquire heart tissue samples for SAV3 RT-qPCR. Heart ventricle is severely damaged during SAV3 viral infection (more severely than pancreas tissue) and heart damage is routinely assessed as an indicator of the severity of the disease^7,11,23^. Samples were preserved in RNAlater (per manufacturer’s specifications, Ambion). Samples from fish dying during the challenge trial before the sampling time points were not used for RT-qPCR. The number of samples used for SAV3 RT-qPCR and mRNA-seq is shown in Table 6.

**Table 6:** Numbers of animals sampled for SAV3 RT-qPCR.

gEBV	Challenge model	Naïve (W0)	W4	W10
High	IP	67 (15)	140 (5)	140 (5)
	CH		141 (5)	123 (5)
Low	IP	68 (15)	116 (5)	109 (5)
	CH		125 (5)	85 (5)
Total		135 (30)	522 (20)	457 (20)

Numbers of samples used for mRNA-seq in parentheses. Salmon with high or low estimated genomic breeding values (gEBV) were infected for the challenge test trials by intraperitoneal injection (IP) or cohabitation with infected fish (CH). Testing occurred one day before infection (W0), 4 weeks post-infection (W4) or 10 weeks post-infection (W10).

### RNA extraction and RT-qPCR

RNA isolation and quantification were performed as described previously^33^. The RT-qPCR was performed in duplicate using a QuantStudio 5 instrument (Applied Biosystems, Thermo Fisher Scientific). The 20µL total volume reactions consisted of 10µL Power SYBR Green PCR Master Mix (Applied Biosystems), 0.6 µL 10 µM forward and reverse primers, 8 µL 1:40 diluted cDNA. The cycling profile was 2 min at 50 °C and 10 min at 95 °C, followed by 40 cycles at 95 °C for 15 s and 60 °C for 60 s. A no-template (water) control was included and a melting curve analysis was performed to verify the measurement of a single specific product. QuantStudio Design & Analysis Software (Applied Biosystems, Thermo Fisher Scientific) was used for data collection and analysis of cycle threshold (*C*_*t*_) values. Primers used for RT-qPCR of salmonid alphavirus were as follows: forward CCGGCCCTGAACCAGTT and reverse GTAGCCAAGTGGGAGAAAGCT^24^. Results are presented as—*C*_*t*_ normalized by RNA concentration.

### Phenotypes

At the end of lab-based analysis and the challenge tests, two phenotypic records were obtained as a measure of resistance against SAV3, survival status (dead/alive, ${P}_{DA}$) and *C*_*t*_ values representing viral load. Individuals that survived until W10 were considered to be survivors.

### Statistical analyses

The heritability of ${P}_{DA}$ and *C*_*t*_ were estimated by ASReml 4.0^68^ using a genomic (${\varvec{G}}$) relationship matrix with the following linear mixed model:

$${\varvec{y}}={\varvec{\mu}}+{\varvec{Z}}{\varvec{u}}+{\varvec{e}}, $$

where ${\varvec{y}}$ is a vector of ‘n’ records on ${P}_{DA}$ and/or $Ct$ values, ${\varvec{\mu}}$ is an overall mean, ${\varvec{u}}$ is a vector of additive genetic effects distributed as ${\varvec{u}}\sim {\varvec{N}}\left(0,{\varvec{G}}{{\varvec{\sigma}}}_{{\varvec{u}}}^{2}\right),$ where ${\sigma }_{u}^{2}$ is the additive genetic variance, ${\varvec{G}}$ is a genomic relationship matrix; ${\varvec{Z}}$ is the corresponding incidence matrix to additive effects, and ${\varvec{e}}$ is the vector of random residual effects with ${\varvec{e}}\sim {\varvec{N}}(0,{\varvec{I}}{{\varvec{\sigma}}}_{{\varvec{e}}}^{2})$. The genomic relationship matrix was constructed using the VanRaden^69^ method as $\frac{{ZZ}^{^{\prime}}}{2*{\sum }_{i=1}^{Nsnp}{p}_{i}(1-{p}_{i})};$ where ${p}_{i}$ is the allele frequency of second allele and $Nsnp$ is the total number of SNP markers. $Z$ is a centered genotype matrix which is equal to ${m}_{ji}-2{p}_{i}$; where ${m}_{ji}$ is the genotype of marker $i$ on animal $j$. The marker genotypes are coded as 0, 1 and 2 representing the homozygote for the first allele, heterozygote, and the homozygote for the second allele, respectively. Heritability (narrow sense) was estimated as the ratio of additive genetic variance to total phenotypic variance by running univariate analyses.

The data was analysed separately for each challenge test as well as collectively including information from both challenge tests. In the collective analysis, challenge model type (IP and CH) was fitted as a fixed effect. Bivariate analysis including both phenotypes in the model was run to determine the genetic correlation between ${P}_{DA}$ and $Ct$ using full dataset (W4 and W10 sampling) covering each of the challenge condition (CH and IP).

### Genome-wide association analysis (GWAS)

A genome wide association analysis was performed using a linear mixed model equation on both ${P}_{DA}$ (binary trait) and ${C}_{t}$ values at W4. However, in this manuscript GWAS results will focus mainly on *C*_*t*_ values because the aim of this study was to evaluate *C*_*t*_ as a trait for testing and selection. Hence, GWAS analysis was performed on *C*_*t*_ values obtained on IP tested individuals and CH tested individuals separately. The model applied for GWAS was the same as described above, however an additional variable was added to estimate marker effects. The GCTA program^70^ with “-mlma-loco” function was used to detect marker ~ trait associations. This approach estimated SNP effects by accounting for additive genetic variance captured by all the markers at chromosomes other than the SNP containing chromosome.

SNPs were considered genome wide significant when they exceeded the Bonferroni threshold for multiple testing (alpha = 0.05) of $0.05/tg$, where $tg$ = 54,282 (total number of SNPs genome-wide) and graded as chromosome-wide significant when Bonferroni threshold for multiple testing surpassed (alpha = 0.05) of $0.05/tc$, where $tc$ = 1,871 (average number of SNPs per chromosome). The genome-wide significant threshold used in this study was considered to be ${P\le 9.21 \times 10 }^{-7}$ which is equivalent to $-{log}_{10}\left(P\right)=6.04$, while chromosome-wide significant threshold was ${P\le 2.67 \times 10 }^{-5}$ which is equal to $-{log}_{10}\left(P\right)=4.57$

The distribution of observed vs. expected *p*-values was plotted as a quantile–quantile (q–q plot) plot, and the inflation factor (lambda, λ) was calculated using following equation

$$lambda \left(\uplambda \right)=\frac{median \left({\chi }^{2}\right)}{0.456}$$

The proportion of variance explained by the top SNPs (*Var*_*SNP*_) was estimated as

$${Var}_{SNPi}=2{p}_{i}{q}_{i}{\alpha }_{i}^{2}$$

where ${p}_{i}$ is the observed allele frequency for the first allele, ${q}_{i}$ is the allele frequency of the alternative allele and ${\alpha }_{i}^{2}$ is the square of the marker effect for the *i*th SNP marker^71^.

### Further SNP detection using mRNA-seq data

Additional SNPs for eQTL detection were identified from the RNA sequencing data using samtools v1.6 software^72^ and genotypes for these additional SNPs were obtained from the RNA sequences of individual samples. PCR duplicates, reads with mapping quality < 20 and bases with phred quality scores < 20 were excluded from analysis. SNPs occurring within 5 bp of an indel, with quality < 20, MAF < 0.10 or less than 4 reads supporting the alternative allele were discarded. SNPs were annotated using SNPeff software^73^.

### Differential gene expression and eQTL detection

The differential expression of genes mapping to the QTL regions was assessed by mRNA-seq at time points W0, W4 and W10 using data and methods described by^33^ for a subset of the animals that were genotyped (Table 6). In short, mRNA-seq was performed on an Illumina HiSeq 3000 platform (6 lanes for the 70 libraries and 150 bp paired-end reads).

Gene expression FPKM values were used for the expression-QTL (eQTL) analysis, considering only those genes showing FPKM > 5 in any of the experimental groups. SNP genotype data (from the NOFSAL3 SNP array and from mRNA-seq identified SNPs) were used. The eQTL analysis was performed using R/MatrixEQTL v2.2.^74^. Two variables, time point of sampling and challenge test model, were fitted as covariates in the model for analysis. The effect of a SNP on gene expression was considered to be in cis if the distance between gene and SNP was < 500 kb. eQTL associations showing FDR corrected *p*-values < 0.05 were considered significant. For those SNPs showing at least one significant trans-eQTL (FDR *p*-value < 0.05) further SNP—gene expression associations were considered using a relaxed nominal *p*-value threshold of 0.01.

## Supplementary information

Supplementary file1 (PDF 412 kb)

## Data Availability

All raw and processed sequence data generated in this study have been deposited in the NCBI Short Read Archive (https://www.ncbi.nlm.nih.gov/sra) under BioProject ID PRJNA543940. GWAS summary statistics data is available in Table 4.
