# Supplementary material for: Quantitative trait loci and genes associated with salmonid alphavirus load in Atlantic salmon: implications for pancreas disease resistance and tolerance
Source: Sci Rep. 2020 Jun 25;10:10393. doi: 10.1038/s41598-020-67405-8 (PMC7316828; doi:10.1038/s41598-020-67405-8)
Supplement: Supplementary file 1 — Supplementary file1 (PDF 412 kb) [file 41598_2020_67405_MOESM1_ESM.pdf]

Supplementary Information

Quantitative trait loci and genes associated with salmonid alphavirus load in Atlantic salmon -Implications for pancreas disease resistance and tolerance

M. L. Aslam[1], D. Robledo[2], A. Krasnov[1], H. K. Moghadam[3], B. Hillestad[3], R. D. Houston[2], M. Baranski[4], S. Boison[4] and N. A. Robinson[15*]

Supplemental Figure S1. Quantile-quantile (q-q) plot for observed and expected genome wide –log$_{10}$ P-values under the CH model.

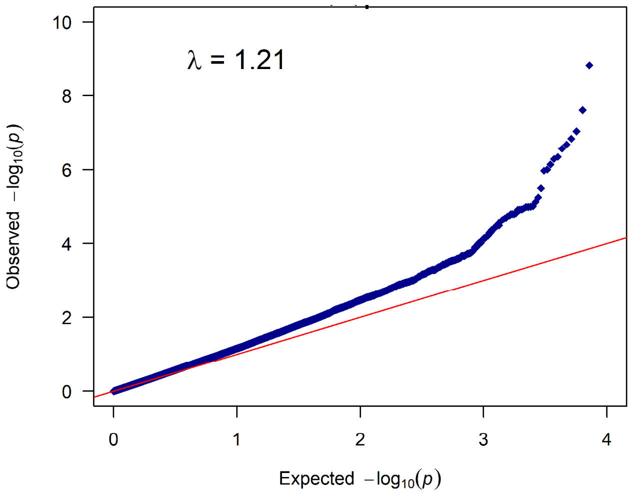

Supplemental table S1. Genes associated with eQTL for viral load on Ssa03. *492 genes associated with SNP AX-88225911 and 68 genes associated with SNP AX-98325250 (and AX-98325256) are listed. Chromosome (chr), position (bases for gene start and gene end), test statistics (including false discovery rate, FDR) and annotation are shown.*

SNP	gene	gene_chr	gene_start	gene_end	beta	t-stat	p-value	FDR	Annotation
AX-88225911	gene57637	jcf1000235881_0_0	4869	5879	18.60950216	6.723074383	5.02E-09	0.008497461	Ubiquitin-like protein
AX-88225911	gene15718	ssa07	20585724	20591265	55.70807509	6.168049073	4.73E-08	0.060102622	Probable E3 ubiquitin-protein ligase RNF144A-A
AX-88225911	gene14275	ssa06	42361507	42408317	2.443993296	5.983086334	9.91E-08	0.100651519	microtubule-associated protein tau-like%2C transcript variant X1
AX-88225911	gene48819	ssa26	29803896	29809376	1.699680186	4.690154003	1.42E-05	1	uncharacterized protein KIAA0355-like
AX-88225911	gene47534	ssa25	17530836	17572988	4.491881923	4.649906803	1.64E-05	1	SH3 domain-binding protein 4
AX-88225911	gene9556	ssa04	34188014	34216340	2.115609808	4.60829242	1.91E-05	1	pro-neuregulin-2%2C membrane-bound isoform-like
AX-88225911	gene28593	ssa13	52592907	52599169	4.78141016	4.595794696	2.00E-05	1	protein sprouty homolog 4-like
AX-88225911	gene10407	ssa04	71565930	71573154	6.744306639	4.563344088	2.25E-05	1	protein sprouty homolog 4-like
AX-88225911	gene15956	ssa07	26437254	26564283	2.936850429	4.474510206	3.10E-05	1	protein Shroom2-like%2C transcript variant X1
AX-88225911	gene11965	ssa05	56864903	56871943	61.95297407	4.445084095	3.44E-05	1	Cathepsin K
AX-88225911	gene71149	jcf1000815095_0_0	206	10786	39.85564882	4.325105009	5.27E-05	1	uncharacterized LOC106594285%2C transcript variant X1
AX-88225911	gene22930	ssa11	16122757	16130210	34.53668285	4.317786295	5.41E-05	1	cartilage intermediate layer protein 1-like%2C transcript variant X1
AX-88225911	gene71150	jcf1000815095_0_0	206	10786	10.88091321	4.255031167	6.74E-05	1	probable E3 ubiquitin-protein ligase RNF144A-A
AX-88225911	gene48889	ssa26	32966968	32998365	1.169763933	4.249344699	6.88E-05	1	uncharacterized LOC106587654%2C transcript variant X1
AX-88225911	gene43072	ssa21	19863821	19867494	14.15899165	4.138127148	0.000101307	1	FK506 binding protein 7
AX-88225911	gene34492	ssa16	20603741	20673034	2.203448475	4.07346476	0.000126562	1	inactive serine protease PAMR1-like
AX-88225911	gene14432	ssa06	50775190	50830286	2.182548648	4.023163512	0.000150301	1	arf-GAP with SH3 domain%2C ANK repeat and PH domain-containing protein 2-like
AX-88225911	gene23033	ssa11	20227837	20268885	2.054322197	4.015521655	0.000154264	1	cingulin-like 1
AX-88225911	gene13785	ssa06	29245633	29309711	1.234396864	3.996805057	0.000164398	1	E3 ubiquitin-protein ligase SMURF2-like
AX-88225911	gene2246	ssa01	117082960	117087378	7.163453168	3.991112644	0.000167605	1	dentin sialophosphoprotein-like
AX-88225911	gene64064	jcf1000511962_0_0	84	1796	3.285467085	3.948318943	0.000193718	1	uncharacterized LOC106591807
AX-88225911	gene39831	ssa19	33128555	33271906	5.138184429	3.923005977	0.00021096	1	nidogen-1-like%2C transcript variant X1
AX-88225911	gene21913	ssa10	79735097	79866077	1.972879437	3.918056795	0.000214499	1	breast cancer anti-estrogen resistance protein 1-like%2C transcript variant X1
AX-88225911	gene48611	ssa26	22018607	22022988	-7.00790996	-3.91253565	0.000218514	1	uncharacterized LOC106587429
AX-88225911	gene11812	ssa05	53074016	53079233	10.21908695	3.908425576	0.00022155	1	uncharacterized LOC106605155%2C transcript variant X1
AX-88225911	gene26334	ssa12	53505193	53510150	1.80179823	3.907690842	0.000222097	1	matrix metalloproteinase-19-like
AX-88225911	gene7567	ssa03	64774491	64787861	36.88946611	3.903482583	0.000225255	1	CD9 molecule
AX-88225911	gene17363	ssa08	21402632	21424229	2.629890445	3.896813616	0.000230348	1	C-type lectin domain family 4 member F-like%2C transcript variant X1
AX-88225911	gene1172	ssa01	54862640	54881594	6.789499439	3.895919364	0.000231039	1	glutamate dehydrogenase%2C mitochondrial-like
AX-88225911	gene51572	ssa28	39561525	39599809	193.1323809	3.887902833	0.000237326	1	tRNA-Ser
AX-88225911	gene4086	ssa02	23783793	23795107	15.83041908	3.887813169	0.000237397	1	cathepsin K-like
AX-88225911	gene36084	ssa16	86503201	86510822	1.598867184	3.846791604	0.000272229	1	ADP-ribosylation factor-like protein 6
AX-88225911	gene23269	ssa11	29828436	29842429	-1.71109967	-3.83669978	0.000281521	1	E3 ubiquitin-protein ligase AMFR%2C transcript variant X1
AX-88225911	gene62243	jcf1000432174_0_0	502	1607	6.641637813	3.820516068	0.000297059	1	glutamate dehydrogenase%2C mitochondrial-like
AX-88225911	gene11953	ssa05	56519890	56532046	6.524731666	3.804813084	0.000312919	1	nestin-like%2C transcript variant X1
AX-88225911	gene44153	ssa22	21122245	21207416	6.329185664	3.7938242	0.000324496	1	calcium/calmodulin-dependent protein kinase type 1-like%2C transcript variant X1
AX-88225911	gene30349	ssa14	28310112	28337903	5.825704384	3.79357574	0.000324763	1	cadherin-4-like
AX-88225911	gene3964	ssa02	20427506	20430769	4.889422825	3.774148067	0.000346258	1	Golgi-associated plant pathogenesis-related protein 1-like%2C transcript variant X1
AX-88225911	gene18486	ssa09	51909769	51941596	6.659333124	3.7602428	0.000362472	1	apelin

AX-88225911	gene43596	ssa21	48681966	48696230	7.062355743	3.750613248	0.000374123	1	major histocompatibility complex class I-related gene protein-like
AX-88225911	gene30525	ssa14	34162787	34171481	8.904475058	3.73374955	0.000395394	1	myeloid-associated differentiation marker homolog
AX-88225911	gene10731	ssa05	4707123	4750868	4.735479067	3.725758988	0.000405872	1	synaptopodin-like
AX-88225911	gene18978	ssa09	71588027	71640363	7.20637331	3.705902558	0.00043307	1	fibroblast growth factor receptor-like 1%2C transcript variant X1
AX-88225911	gene62060	jcf1000423384_0_0	10693	11130	17.25217131	3.665654274	0.00049363	1	Ubiquitin-conjugating enzyme E2 variant 1
AX-88225911	gene33856	ssa15	91208097	91222997	-3.14297035	-3.66406877	0.000496174	1	isocitrate dehydrogenase [NAD] subunit gamma%2C mitochondrial-like%2C transcript variant X1
AX-88225911	gene39010	ssa18	60655988	60661747	101.8714309	3.659147145	0.00050415	1	plasminogen activator inhibitor 1-like
AX-88225911	gene36931	ssa17	27313349	27322840	8.366712259	3.629417034	0.000554993	1	ly6/PLAUR domain-containing protein 6-like%2C transcript variant X1
AX-88225911	gene43980	ssa22	12272094	12281619	7.033621197	3.626260368	0.00056067	1	tumor necrosis factor receptor superfamily member 1B-like%2C transcript variant X1
AX-88225911	gene22315	ssa10	99802106	99814774	17.3357113	3.61779349	0.00057617	1	dickkopf-related protein 3-like
AX-88225911	gene43591	ssa21	48422418	48462209	4.423092004	3.613829574	0.000583566	1	Glycophorin-C
AX-88225911	gene24232	ssa11	73677190	73682070	117.1921856	3.609196393	0.000592325	1	procollagen C-endopeptidase enhancer 2-like
AX-88225911	gene36638	ssa17	18383186	18399153	3.439503484	3.608549927	0.000593557	1	uncharacterized LOC106575549%2C transcript variant X1
AX-88225911	gene40250	ssa19	51990100	52028923	4.273440345	3.601066383	0.000607998	1	angiotensin-converting enzyme-like
AX-88225911	gene7466	ssa03	62451522	62460089	2.089556431	3.597080865	0.000615825	1	Interleukin-4 receptor alpha chain
AX-88225911	gene45278	ssa23	9623136	9931011	8.750332851	3.593563527	0.000622813	1	cAMP-specific 3'%2C5'-cyclic phosphodiesterase 4B-like%2C transcript variant X1
AX-88225911	gene47131	ssa24	41009562	41020152	75.83756234	3.587321623	0.000635398	1	C-C motif chemokine 19-like
AX-88225911	gene49923	ssa27	11968026	11977886	3.559989352	3.554941316	0.000704664	1	uncharacterized LOC106588333%2C transcript variant X1
AX-88225911	gene30836	ssa14	45282952	45296183	-6.68640396	-3.54538721	0.000726435	1	peroxisomal trans-2-enoyl-CoA reductase
AX-88225911	gene26287	ssa12	52300651	52322213	-0.88946014	-3.53864266	0.000742187	1	protein SYS1 homolog%2C transcript variant X1
AX-88225911	gene1579	ssa01	79498027	79512120	8.692777676	3.531190485	0.00075997	1	peripheral myelin protein 22-like
AX-88225911	gene12237	ssa05	65511549	65528667	4.413281391	3.528325076	0.000766914	1	collagen triple helix repeat-containing protein 1-like%2C transcript variant X1
AX-88225911	gene19430	ssa09	95116162	95140489	4.634822749	3.525693514	0.000773345	1	stress-associated endoplasmic reticulum protein 1-like
AX-88225911	gene23583	ssa11	41282455	41287381	4.567322974	3.524308705	0.000776749	1	transmembrane 6 superfamily member 1
AX-88225911	gene51311	ssa28	25321179	25322528	10.64363039	3.519292157	0.000789202	1	MARVEL domain-containing protein 1-like
AX-88225911	gene26376	ssa12	55068173	55073157	20.61423862	3.514776511	0.000800573	1	uncharacterized LOC106565475
AX-88225911	gene21505	ssa10	54561237	54738510	0.975211166	3.510027842	0.000812699	1	ankyrin repeat and BTB (POZ) domain containing 2%2C transcript variant X1
AX-88225911	gene989	ssa01	45034991	45048763	6.425336965	3.497963402	0.000844294	1	fos-related antigen 2-like%2C transcript variant X1
AX-88225911	gene21170	ssa10	36143857	36213028	2.76787172	3.490482718	0.000864466	1	cadherin-2-like
AX-88225911	gene33957	ssa15	94963567	94979084	-2.58185174	-3.48858837	0.000869647	1	tumor necrosis factor receptor superfamily member 5-like%2C transcript variant X1
AX-88225911	gene31774	ssa14	85491866	85503687	9.595255531	3.486666132	0.000874933	1	wiskott-Aldrich syndrome protein family member 2-like
AX-88225911	gene32555	ssa15	33069572	33091027	1.527968989	3.486652079	0.000874972	1	Golgi-associated PDZ and coiled-coil motif-containing protein-like%2C transcript variant X1
AX-88225911	gene13964	ssa06	33802332	33807836	8.566142951	3.470288094	0.00092123	1	interferon-induced very large GTPase 1-like
AX-88225911	gene29050	ssa13	79026894	79047795	2.620133275	3.462890937	0.000942892	1	Long-chain-fatty-acid--CoA ligase 4
AX-88225911	gene47970	ssa25	38165951	38176542	5.73618914	3.457252621	0.000959727	1	uncharacterized LOC106586715%2C transcript variant X1
AX-88225911	gene43687	ssa21	53794136	53858372	2.211654004	3.453975066	0.000969643	1	integrin%2C alpha V
AX-88225911	gene30112	ssa14	17138247	17249913	1.786677231	3.449979763	0.000981862	1	glypican-1-like
AX-88225911	gene71124	jcf1000813969_0_0	9218	11594	2.676880172	3.448496186	0.000986437	1	uncharacterized LOC106592919
AX-88225911	gene42297	ssa20	60374978	60385011	1.391704879	3.445854594	0.000994632	1	junctional adhesion molecule 3%2C transcript variant X1
AX-88225911	gene32806	ssa15	43856482	43885412	2.21958109	3.443551923	0.001001828	1	uncharacterized protein C14orf132-like
AX-88225911	gene18583	ssa09	57250288	57254178	1.965392967	3.442908185	0.001003849	1	proteinase-activated receptor 4-like
AX-88225911	gene40285	ssa19	53074305	53086161	2.729258094	3.436965069	0.001022686	1	uncharacterized LOC106579236
AX-88225911	gene11477	ssa05	35555828	35652675	1.540124105	3.433216513	0.001034739	1	protocadherin alpha-C2-like%2C transcript variant X1
AX-88225911	gene6159	ssa03	12543541	12546292	8.629557898	3.42764118	0.001052915	1	growth arrest and DNA damage-inducible protein GADD45 alpha-like
AX-88225911	gene45468	ssa23	21301183	21318544	-2.95169208	-3.42657439	0.001056427	1	carbonyl reductase 4

AX-88225911	gene17991	ssa09	25211291	25239564	7.171289509	3.424484908	0.001063338	1	sacsin-like%2C transcript variant X1
AX-88225911	gene28874	ssa13	70708316	70813127	3.040586419	3.408660283	0.001117084	1	drebrin-like%2C transcript variant X1
AX-88225911	gene1413	ssa01	71491055	71530954	82.20109773	3.40844437	0.001117834	1	cysteine-rich protein 2-like%2C transcript variant X1
AX-88225911	gene40030	ssa19	44015573	44017768	173.7945687	3.39080254	0.001180817	1	uncharacterized LOC106578920
AX-88225911	gene30121	ssa14	18283740	18310722	26.73471538	3.387595597	0.00119262	1	integrin beta-1-like%2C transcript variant X1
AX-88225911	gene48588	ssa26	21100545	21165512	1.336578402	3.386717927	0.00119587	1	transcription factor 12-like%2C transcript variant X1
AX-88225911	gene22037	ssa10	85899762	85927615	1.640710721	3.381887708	0.001213905	1	hepatocyte growth factor-like
AX-88225911	gene53221	jcf1000042214_0_0	549	6533	2.490733583	3.380596625	0.001218769	1	mpv17-like protein
AX-88225911	gene28634	ssa13	55680224	55697841	2.42587882	3.379371478	0.001223401	1	serine/threonine-protein phosphatase 2A catalytic subunit alpha isoform-like%2C transcript variant X1
AX-88225911	gene2269	ssa01	117808419	117851907	1.658087945	3.366428608	0.001273367	1	retinoic acid induced 14%2C transcript variant X1
AX-88225911	gene30967	ssa14	51033367	51050333	9.887960867	3.362241079	0.001289941	1	BOLA class I histocompatibility antigen%2C alpha chain BL3-7-like
AX-88225911	gene37521	ssa17	48525511	48527477	43.62216013	3.36122044	0.001294012	1	Ig kappa chain V region 3381-like
AX-88225911	gene2678	ssa01	135337320	135340733	4.00294214	3.35928916	0.001301747	1	coagulation factor II (thrombin) receptor
AX-88225911	gene30626	ssa14	37654548	37657931	6.826522823	3.356093172	0.001314644	1	C2 calcium-dependent domain-containing protein 4C-like
AX-88225911	gene14137	ssa06	38188127	38194423	2.363754671	3.355950179	0.001315224	1	synaptonemal complex protein SC65-like
AX-88225911	gene4081	ssa02	23714703	23717278	22.00322625	3.338322786	0.001388581	1	induced myeloid leukemia cell differentiation protein Mcl-1 homolog
AX-88225911	gene49850	ssa27	10153895	10157742	17.08479929	3.338055362	0.001389724	1	proteasome subunit beta type-8-like%2C transcript variant X1
AX-88225911	gene50674	ssa27	39986382	40002565	2.141415195	3.328446841	0.00143135	1	nucleoporin like 2
AX-88225911	gene52217	ssa29	39236913	39363189	7.26841113	3.326774113	0.001438716	1	procollagen-lysine%2C2-oxoglutarate 5-dioxygenase 2-like%2C transcript variant X1
AX-88225911	gene22520	ssa10	108506471	108519582	6.594591146	3.312208374	0.001504383	1	ETS domain-containing protein Elk-3-like
AX-88225911	gene72089	jcf1000856931_0_0	5	55	2.110770371	3.311194005	0.00150906	1	N-acetylglucosamine-6-sulfatase-like
AX-88225911	gene31386	ssa14	66777079	66781049	5.91609543	3.310138626	0.00151394	1	endothelin-2-like
AX-88225911	gene50325	ssa27	23145824	23179275	1.491785684	3.302624517	0.001549119	1	syndecan 3%2C transcript variant X1
AX-88225911	gene46328	ssa24	9279796	9355922	5.479300366	3.302133336	0.001551445	1	lysyl oxidase homolog 2B-like%2C transcript variant X1
AX-88225911	gene14136	ssa06	38182007	38187268	5.297579583	3.300406478	0.00155965	1	peptidyl-prolyl cis-trans isomerase FKBP10-like
AX-88225911	gene45300	ssa23	11129336	11141708	3.674885331	3.294149164	0.001589722	1	uridine-cytidine kinase 2-A%2C transcript variant X1
AX-88225911	gene27453	ssa13	6141900	6149211	47.07640156	3.292790805	0.001596322	1	matrix metalloproteinase-9-like
AX-88225911	gene28201	ssa13	36110994	36125992	-3.85096887	-3.29026381	0.001608669	1	carboxy-terminal domain RNA polymerase II polypeptide A small phosphatase 2-like
AX-88225911	gene67532	jcf1000658548_0_0	4792	9427	10.87334481	3.285239134	0.001633487	1	uncharacterized LOC106593137%2C transcript variant X1
AX-88225911	gene18439	ssa09	48189270	48205393	4.950485499	3.283819512	0.001640564	1	arginase-2%2C mitochondrial-like
AX-88225911	gene38757	ssa18	53454158	53470782	31.73188322	3.283274071	0.00164329	1	RING finger protein 182
AX-88225911	gene13096	ssa06	9378781	9397834	22.05747251	3.283085766	0.001644233	1	metalloproteinase inhibitor 2-like
AX-88225911	gene52976	jcf1000031470_0-2742_unknown_0_832	238	1376	5.642020579	3.281832857	0.001650515	1	calponin-3-like
AX-88225911	gene48529	ssa26	18453987	18520468	3.050039947	3.276120042	0.001679451	1	eukaryotic translation initiation factor 4 gamma 2-like
AX-88225911	gene40843	ssa19	79456072	79475233	4.021962794	3.275518868	0.001682523	1	retinol dehydrogenase 10 (all-trans)
AX-88225911	gene48891	ssa26	33052123	33062297	1.654026771	3.274569589	0.001687386	1	cytoplasmic polyadenylation element-binding protein 1-like%2C transcript variant X1
AX-88225911	gene24607	ssa11	83637619	83655351	6.862387582	3.268006035	0.001721369	1	glutamine--fructose-6-phosphate aminotransferase [isomerizing] 2-like
AX-88225911	gene38869	ssa18	56559644	56566410	43.32448191	3.26542592	0.001734903	1	transmembrane protein 88-like
AX-88225911	gene51696	ssa29	12474810	12499543	2.392679161	3.265118666	0.001736521	1	tripartite motif-containing protein 16-like
AX-88225911	gene7394	ssa03	60362178	60442821	1.522731032	3.261203337	0.001757269	1	inactive phospholipase C-like protein 2%2C transcript variant X1
AX-88225911	gene51276	ssa28	21931889	21937283	140.1983288	3.261145844	0.001757575	1	ankyrin repeat domain-containing protein 1-like
AX-88225911	gene28044	ssa13	29364450	29367133	2.71325577	3.260444855	0.001761315	1	tumor necrosis factor receptor superfamily%2C member 6b%2C decoy%2C transcript variant X1
AX-88225911	gene16450	ssa07	46696867	46700830	1.729477422	3.259416587	0.001766814	1	cytoskeleton-associated protein 4-like
AX-88225911	gene20879	ssa10	22513890	22619420	1.041453968	3.255986835	0.001785274	1	long-chain-fatty-acid--CoA ligase ACSBG2-like%2C transcript variant X1
AX-88225911	gene50614	ssa27	36791862	36800467	1.344934797	3.255171048	0.001789691	1	tRNA selenocysteine 1-associated protein 1-like%2C transcript variant X1

AX-88225911	gene5292	ssa02	63705770	63712275	4.229478305	3.251563593	0.001809348	1	GTPase IMAP family member 4-like%2C transcript variant X1
AX-88225911	gene77375	jcf1001073308_0_0	52	45937	-1.54988425	-3.25061731	0.001814538	1	LIM domain-binding protein 1%2C transcript variant X4
AX-88225911	gene4359	ssa02	33596518	33603247	2.326373194	3.2460322	0.001839884	1	protein FAM46A-like
AX-88225911	gene25158	ssa12	8352041	8354399	16.5387281	3.240417793	0.001871372	1	lipopolysaccharide-induced tumor necrosis factor-alpha factor homolog
AX-88225911	gene36281	ssa17	8151585	8157698	1.698912339	3.240148645	0.001872894	1	ras-related protein M-Ras-like
AX-88225911	gene29728	ssa14	4017655	4025087	3.358391311	3.239996055	0.001873757	1	muscle-related coiled-coil protein-like
AX-88225911	gene44985	ssa22	55931880	55995590	1.277586743	3.234353054	0.001905954	1	dystroglycan 1 (dystrophin-associated glycoprotein 1)%2C transcript variant X1
AX-88225911	gene14309	ssa06	43793004	43801400	-2.14717308	-3.23386018	0.001908791	1	peroxisomal membrane protein PMP34-like%2C transcript variant X1
AX-88225911	gene11586	ssa05	40705910	40707544	4.815262132	3.230802232	0.001926479	1	heterogeneous nuclear ribonucleoprotein A0-like
AX-88225911	gene2625	ssa01	132206512	132253431	1.049512237	3.226630662	0.001950858	1	niban-like protein 1
AX-88225911	gene69913	jcf1000759333_0-0_0_0	5376	7803	3.132109234	3.22409819	0.001965798	1	ras homolog family member H%2C transcript variant X1
AX-88225911	gene72864	jcf1000889505_0_0	3232	6563	-3.29617155	-3.22168303	0.001980147	1	bromodomain-containing protein 9-like
AX-88225911	gene29327	ssa13	90016975	90041610	-9.61430186	-3.22036947	0.001987992	1	uncharacterized LOC106568152
AX-88225911	gene51449	ssa28	31620818	31678838	20.75144211	3.216002203	0.002014285	1	vinculin-like%2C transcript variant X1
AX-88225911	gene29471	ssa13	95219181	95311698	3.361561773	3.212824411	0.002033622	1	integrin alpha-1-like%2C transcript variant X1
AX-88225911	gene3798	ssa02	14165094	14189790	80.15476796	3.2094215	0.002054522	1	glyceraldehyde-3-phosphate dehydrogenase
AX-88225911	gene23263	ssa11	29650370	29671526	19.34729064	3.204210852	0.002086915	1	matrix metalloproteinase 2
AX-88225911	gene39320	ssa18	70090338	70099364	0.90200063	3.197533274	0.002129128	1	PC4 and SFRS1-interacting protein-like%2C transcript variant X1
AX-88225911	gene4248	ssa02	27776375	27776797	585.0543594	3.193802545	0.00215306	1	uncharacterized LOC106582879
AX-88225911	gene35534	ssa16	69599944	69652863	1.517924681	3.193386381	0.002155745	1	E3 ubiquitin-protein ligase Midline-1-like%2C transcript variant X1
AX-88225911	gene4504	ssa02	40862522	40872948	5.612464287	3.192619849	0.002160699	1	3-oxo-5-alpha-steroid 4-dehydrogenase 2
AX-88225911	gene48983	ssa26	35488111	35489575	2.209357205	3.185306115	0.002208507	1	Ig kappa chain V-II region 7S34.1-like isoform X1
AX-88225911	gene27587	ssa13	10479216	10481126	-4.7255629	-3.18157005	0.002233309	1	ubiquitously-expressed%2C prefoldin-like chaperone
AX-88225911	gene21624	ssa10	65889945	65916462	13.78575979	3.181550761	0.002233438	1	protein disulfide isomerase family A%2C member 3
AX-88225911	gene57740	jcf1000239655_0_0	20655	24890	2.288065747	3.177844785	0.0022583	1	uncharacterized LOC106592021
AX-88225911	gene49930	ssa27	12039117	12078644	0.991138837	3.176041635	0.00227049	1	protein disulfide-isomerase A3-like%2C transcript variant X1
AX-88225911	gene49950	ssa27	12589202	12597227	26.18554177	3.175352508	0.002275165	1	S100-A14
AX-88225911	gene6553	ssa03	30547238	30565036	2.231141507	3.171500629	0.002301463	1	microtubule-associated protein RP/EB family member 2-like%2C transcript variant X1
AX-88225911	gene5156	ssa02	61198092	61237005	3.076327566	3.168521585	0.002321997	1	transmembrane channel-like protein 6%2C transcript variant X1
AX-88225911	gene1747	ssa01	92828267	92841689	-0.94003608	-3.16260051	0.00236332	1	serine/threonine-protein kinase ICK-like%2C transcript variant X1
AX-88225911	gene43953	ssa22	11558836	11578746	3.792594702	3.160448463	0.002378508	1	ras association domain-containing protein 1-like%2C transcript variant X1
AX-88225911	gene16103	ssa07	35380514	35406989	8.273439674	3.156333541	0.002407806	1	cAMP-responsive element-binding protein 3-like protein 2
AX-88225911	gene22323	ssa10	100072418	100178132	11.1522502	3.155011845	0.002417287	1	spondin-1-like
AX-88225911	gene26478	ssa12	58953144	58973350	40.48160021	3.154806	0.002418767	1	ATP-dependent 6-phosphofructokinase%2C muscle type-like
AX-88225911	gene1640	ssa01	84637975	84648121	89.87078223	3.154399616	0.002421691	1	ankyrin repeat domain-containing protein 1-like
AX-88225911	gene29815	ssa14	8762239	8786214	3.015624413	3.146937981	0.002475971	1	tyrosine-protein kinase JAK1-like
AX-88225911	gene11032	ssa05	15804195	15808445	-1.49940979	-3.14593897	0.002483324	1	retinoic acid receptor responder protein 3-like
AX-88225911	gene39134	ssa18	64099595	64120382	1.047199169	3.144695249	0.002492507	1	kinesin light chain 2-like%2C transcript variant X1
AX-88225911	gene48993	ssa26	36179415	36189399	2.29170785	3.137532929	0.002546011	1	cysteinyl-tRNA synthetase
AX-88225911	gene41670	ssa20	37020378	37141827	-1.24862118	-3.13408519	0.002572147	1	COBW domain containing 3%2C transcript variant X1
AX-88225911	gene23034	ssa11	20271580	20291571	0.674505444	3.133765836	0.002574581	1	transcription factor 12%2C transcript variant X1
AX-88225911	gene31627	ssa14	78914910	78969038	6.645528732	3.132934775	0.002580924	1	tubulointerstitial nephritis antigen-like
AX-88225911	gene41000	ssa20	7077184	7086819	15.80398959	3.131173131	0.002594418	1	tetraspanin-3-like%2C transcript variant X1
AX-88225911	gene49186	ssa26	42024204	42028162	25.18945876	3.128520943	0.002614858	1	heat shock factor-binding protein 1-like
AX-88225911	gene42680	ssa20	80568458	80587593	3.916232043	3.126029467	0.002634196	1	small G protein signaling modulator 2-like
AX-88225911	gene7300	ssa03	57919946	57949143	37.13828574	3.122961466	0.002658194	1	eukaryotic translation initiation factor 1

AX-88225911	gene52184	ssa29	35720217	35748903	3.958957132	3.122552034	0.002661411	1	calponin-3-like
AX-88225911	gene23153	ssa11	24035884	24053991	1.629488081	3.121370574	0.002670717	1	AP-2 complex subunit alpha-2%2C transcript variant X1
AX-88225911	gene23529	ssa11	38937816	38940691	3.652372665	3.120024166	0.00268136	1	transmembrane 6 superfamily member 1-like
AX-88225911	gene47631	ssa25	21545724	21621799	0.937590335	3.120016873	0.002681418	1	integrin beta-5-like%2C transcript variant X1
AX-88225911	gene19090	ssa09	77004621	77024526	2.449064126	3.11947791	0.002685689	1	fibronectin type-III domain-containing protein UNQ728/PRO1410 homolog
AX-88225911	gene30858	ssa14	45849210	45867164	6.359527144	3.117095383	0.002704647	1	VAMP (vesicle-associated membrane protein)-associated protein A%2C 33kDa
AX-88225911	gene50582	ssa27	35231632	35239011	2.202030182	3.117064857	0.00270489	1	FUS-interacting serine-arginine-rich protein 1
AX-88225911	gene31180	ssa14	59066600	59094206	7.656900449	3.116456327	0.002709753	1	class I histocompatibility antigen%2C F10 alpha chain-like
AX-88225911	gene3608	ssa02	10141376	10151460	31.1396745	3.114065515	0.002728936	1	receptor tyrosine kinase
AX-88225911	gene40368	ssa19	55795324	55832507	3.835497773	3.109476683	0.002766111	1	peptidyl-prolyl cis-trans isomerase FKBP10-like
AX-88225911	gene37547	ssa17	49153678	49177635	3.852643046	3.100690348	0.002838615	1	poly [ADP-ribose] polymerase 12-like%2C transcript variant X1
AX-88225911	gene37146	ssa17	35296846	35448554	29.21722285	3.096783922	0.002871417	1	Metalloproteinase inhibitor 3
AX-88225911	gene30881	ssa14	46908514	46975575	1.448560957	3.095477087	0.002882469	1	rho GTPase-activating protein 29-like%2C transcript variant X1
AX-88225911	gene51383	ssa28	28252615	28278152	8.52873886	3.087857462	0.002947705	1	hexokinase-1-like%2C transcript variant X1
AX-88225911	gene25505	ssa12	19630840	19632724	26.44639064	3.084343754	0.002978249	1	C-X-C motif chemokine 11-like
AX-88225911	gene21357	ssa10	46118370	46235504	2.020315542	3.078592257	0.003028885	1	serine/threonine-protein kinase Nek7-like%2C transcript variant X1
AX-88225911	gene24348	ssa11	76389385	76437008	1.714295383	3.070256974	0.003103695	1	latent-transforming growth factor beta-binding protein 3-like%2C transcript variant X1
AX-88225911	gene24067	ssa11	65356302	65366443	3.000034003	3.068440836	0.003120222	1	DEAD (Asp-Glu-Ala-Asp) box polypeptide 54
AX-88225911	gene50321	ssa27	23069463	23093234	9.550158884	3.068398879	0.003120605	1	proto-oncogene tyrosine-protein kinase Yrk-like%2C transcript variant X1
AX-88225911	gene3426	ssa02	6238140	6240921	13.27218805	3.067593855	0.003127957	1	protein asteroid homolog 1-like
AX-88225911	gene19288	ssa09	87792447	87819724	2.279035988	3.067568643	0.003128188	1	phospholipase D3-like%2C transcript variant X1
AX-88225911	gene4785	ssa02	48865612	48867386	15.05992386	3.066248801	0.003140279	1	C-X-C motif chemokine 11-like
AX-88225911	gene36244	ssa17	6697996	6728896	1.356696098	3.064154944	0.00315955	1	SH3 domain-binding protein 4-A-like%2C transcript variant X1
AX-88225911	gene28931	ssa13	74919167	74934154	3.270605006	3.063737791	0.003163402	1	B-cell translocation gene 3
AX-88225911	gene14719	ssa06	62161583	62176026	7.6801243	3.057413789	0.003222343	1	adenylosuccinate synthase like 1
AX-88225911	gene33908	ssa15	93301187	93307813	54.49711582	3.053665908	0.003257756	1	keratin%2C type I cytoskeletal 18
AX-88225911	gene37490	ssa17	47898379	47909509	8.267119626	3.053125169	0.003262895	1	Sodium-coupled neutral amino acid transporter 2
AX-88225911	gene15614	ssa07	17586071	17590072	11.65080508	3.049624571	0.003296347	1	uncharacterized LOC106608768%2C transcript variant X1
AX-88225911	gene20696	ssa10	16443572	16457490	6.816764555	3.048397816	0.003308145	1	myeloid-derived growth factor-like
AX-88225911	gene34738	ssa16	31143475	31177432	3.00787993	3.046246427	0.003328932	1	core-binding factor subunit beta%2C transcript variant X1
AX-88225911	gene62647	jcf1000447823_0_0	2	1443	2.467325785	3.04480136	0.003342962	1	transmembrane channel-like protein 6%2C transcript variant X1
AX-88225911	gene19957	ssa09	114989113	115001006	1.99630528	3.040546745	0.00338459	1	hypermethylated in cancer 1 protein-like%2C transcript variant X1
AX-88225911	gene48994	ssa26	36191855	36198630	4.959831057	3.039916949	0.003390793	1	Cysteinyl-tRNA synthetase%2C cytoplasmic
AX-88225911	gene47666	ssa25	24080473	24101174	6.111039129	3.036583006	0.003423807	1	basic leucine zipper and W2 domain-containing protein 1-A-like
AX-88225911	gene21831	ssa10	76228834	76254302	1.356444707	3.035768528	0.003431917	1	septin-7-like%2C transcript variant X1
AX-88225911	gene45768	ssa23	34792834	34796586	4.055241384	3.033046055	0.003459157	1	cyclin-dependent kinase inhibitor 1B-like
AX-88225911	gene68539	jcf1000701251_0_0	1637	11546	2.103749501	3.03085547	0.003481221	1	low density lipoprotein receptor-related protein associated protein 1%2C transcript variant X1
AX-88225911	gene12766	ssa05	77676821	77687920	14.9679538	3.026901621	0.003521378	1	uncharacterized LOC106606114
AX-88225911	gene14121	ssa06	37848177	37853413	3.03466448	3.024722836	0.00354369	1	probable ATP-dependent RNA helicase DHX58
AX-88225911	gene69289	jcf1000732523_0_0	7488	8613	1.768823104	3.024344083	0.003547582	1	uncharacterized LOC106594413
AX-88225911	gene45037	ssa22	59971334	59984614	5.1514327	3.023903496	0.003552114	1	protein shisa-5-like%2C transcript variant X1
AX-88225911	gene294	ssa01	14343363	14354018	20.38565324	3.02291147	0.003562339	1	14-3-3 protein beta/alpha
AX-88225911	gene49824	ssa27	9257912	9287477	14.43918633	3.019424656	0.003598496	1	C type lectin receptor C
AX-88225911	gene40766	ssa19	74288873	74346695	6.480503575	3.015699308	0.003637503	1	myeloid cell surface antigen CD33-like%2C transcript variant X1
AX-88225911	gene26686	ssa12	68929649	68966720	3.314311743	3.014209722	0.00365321	1	MAP kinase-activated protein kinase 3-like
AX-88225911	gene47658	ssa25	22877617	22890059	1.788302804	3.013539932	0.003660293	1	sterile alpha motif domain-containing protein 9-like

AX-88225911	gene9740	ssa04	38539530	38543787	33.16960159	3.012756516	0.003668594	1	procollagen C-endopeptidase enhancer 2-like
AX-88225911	gene10530	ssa04	77724486	77739316	-5.73040339	-3.01249216	0.003671399	1	pterin-4 alpha-carbinolamine dehydratase/dimerization cofactor of hepatocyte nuclear factor 1 alpha (TCF1) 2
AX-88225911	gene23926	ssa11	57914944	57917711	3.998214889	3.010644233	0.003691063	1	proteinase-activated receptor 1-like
AX-88225911	gene13143	ssa06	11581423	11597862	33.27138153	3.009656361	0.003701615	1	sarcalumenin-like%2C transcript variant X2
AX-88225911	gene18299	ssa09	41416775	41418661	9.709477676	3.008174047	0.003717501	1	dual specificity phosphatase 23
AX-88225911	gene18608	ssa09	58671634	58688904	1.424589715	3.008096893	0.00371833	1	fibroblast growth factor 2 (basic)
AX-88225911	gene37505	ssa17	48279156	48291663	1.643251075	3.0042017	0.003760386	1	diphosphoinositol polyphosphate phosphohydrolase 3-beta-like
AX-88225911	gene48153	ssa25	49106153	49197473	2.873038713	3.004196249	0.003760445	1	integrin alpha-V-like
AX-88225911	gene17225	ssa08	18068612	18089997	1.4249857	3.001395358	0.00379096	1	3'-phosphoadenosine 5'-phosphosulfate synthase 1
AX-88225911	gene16293	ssa07	42417658	42430371	3.188548856	2.999545635	0.003811238	1	adenosine deaminase CECR1-A-like
AX-88225911	gene18047	ssa09	27129400	27134433	12.46623563	2.999529187	0.003811419	1	ornithine decarboxylase 1-like
AX-88225911	gene49777	ssa27	7678810	8014319	11.47308367	2.998263221	0.003825356	1	C-type lectin domain family 4 member E-like%2C transcript variant X1
AX-88225911	gene17739	ssa09	16130570	16136718	12.6745058	2.997672845	0.003831871	1	glutaminyl-peptide cyclotransferase-like
AX-88225911	gene20630	ssa10	14560067	14565468	46.27418003	2.99764931	0.003832131	1	uncharacterized LOC106613620
AX-88225911	gene36264	ssa17	7390758	7398812	3.056967537	2.997272488	0.003836296	1	septin-10-like
AX-88225911	gene39807	ssa19	31652261	31746694	0.951531309	2.993369906	0.003879673	1	transmembrane protein 108-like%2C transcript variant X1
AX-88225911	gene3321	ssa02	3311985	3322330	1.53718254	2.990378117	0.003913235	1	toll-like receptor 13
AX-88225911	gene30053	ssa14	15358617	15376187	1.681917652	2.989397718	0.003924291	1	Calponin-3
AX-88225911	gene76340	jcf1001030339_0_0	9	6	1.084499189	2.98688672	0.003952742	1	transcriptional regulator ATRX-like
AX-88225911	gene59458	jcf1000313339_0_0	1259	10484	1.190853941	2.986335858	0.003959009	1	sister chromatid cohesion protein PDS5 homolog A-like
AX-88225911	gene271	ssa01	12140592	12148744	3.71717236	2.985399122	0.003969687	1	heterogeneous nuclear ribonucleoprotein Q%2C transcript variant X1
AX-88225911	gene44858	ssa22	50319827	50321234	45.36107015	2.984220676	0.003983159	1	uncharacterized LOC106583655
AX-88225911	gene37296	ssa17	41821897	41831482	3.53894877	2.982393811	0.004004127	1	6-phosphofructo-2-kinase/fructose-2%2C6-bisphosphatase 3-like%2C transcript variant X1
AX-88225911	gene46535	ssa24	17379547	17391055	3.097607161	2.982145883	0.004006981	1	TRAF-type zinc finger domain-containing protein 1-like
AX-88225911	gene50634	ssa27	37688248	37718451	1.388732625	2.980365996	0.004027522	1	serum response factor-binding protein 1-like
AX-88225911	gene21901	ssa10	79234127	79261776	2.505724699	2.979035147	0.004042944	1	copine-2-like
AX-88225911	gene49848	ssa27	10122007	10149393	381.704463	2.978314096	0.004051323	1	class I histocompatibility antigen%2C F10 alpha chain-like
AX-88225911	gene20845	ssa10	21508960	21513048	1.236624738	2.977406686	0.00406189	1	Secretory carrier-associated membrane protein 4
AX-88225911	gene17808	ssa09	18892788	18900129	10.15993159	2.975885086	0.004079668	1	KH domain-containing%2C RNA-binding%2C signal transduction-associated protein 1%2C transcript variant X1
AX-88225911	gene13095	ssa06	9303996	9308495	1.299654122	2.974140413	0.00410014	1	metalloproteinase inhibitor 2-like
AX-88225911	gene32366	ssa15	24233112	24290080	3.823668057	2.973242844	0.00411071	1	ectonucleotide pyrophosphatase/phosphodiesterase family member 3-like
AX-88225911	gene15839	ssa07	23205703	23213642	22.17233541	2.973183392	0.004111411	1	plasminogen activator inhibitor 1-like
AX-88225911	gene33066	ssa15	59506236	59567700	1.132905686	2.969616007	0.004153676	1	nuclear pore membrane glycoprotein 210-like
AX-88225911	gene14689	ssa06	60891640	61074564	2.760962604	2.96359884	0.00422588	1	enabled homolog (Drosophila)%2C transcript variant X1
AX-88225911	gene38186	ssa18	21501566	21518820	10.05968794	2.961727806	0.004248568	1	prolyl 4-hydroxylase subunit alpha-1-like
AX-88225911	gene3852	ssa02	15565305	15574901	2.412046383	2.960074399	0.004268711	1	platelet-activating factor acetylhydrolase IB subunit gamma-like%2C transcript variant X1
AX-88225911	gene30055	ssa14	15422983	15426979	5.262580056	2.959870419	0.004271202	1	tissue factor-like
AX-88225911	gene34170	ssa16	1165157	1222830	14.70869983	2.952424568	0.004363065	1	CD82 antigen-like%2C transcript variant X1
AX-88225911	gene29978	ssa14	12723645	12731752	10.79618919	2.951022116	0.004380572	1	angiopoietin-related protein 4-like
AX-88225911	gene50341	ssa27	23554769	23658088	3.510385274	2.949486563	0.004399814	1	peptidyl-prolyl cis-trans isomerase FKBP9-like
AX-88225911	gene5691	ssa03	1859543	1891110	2.088851445	2.948832743	0.004408031	1	far upstream element (FUSE) binding protein 1%2C transcript variant X1
AX-88225911	gene49877	ssa27	10648406	10658002	15.49324015	2.948018363	0.004418286	1	class I histocompatibility antigen%2C F10 alpha chain-like%2C transcript variant X1
AX-88225911	gene13448	ssa06	21781703	21788057	-3.08077928	-2.9453582	0.004451937	1	Alkylated DNA repair protein alkB homolog 7
AX-88225911	gene13047	ssa06	7430239	7430614	117.5228437	2.945007028	0.004456397	1	Metalloproteinase inhibitor 2

AX-88225911	gene62203	jcf1000430681_0_0	6	1700	2.441398854	2.94489394	0.004457835		1	alpha-actinin-2-like
AX-88225911	gene37871	ssa18	6289742	6304424	32.2274234	2.944086192	0.004468112		1	PDZ and LIM domain protein 1
AX-88225911	gene19617	ssa09	103077297	103081324	5.889712784	2.94162748	0.004499532		1	TRAF-type zinc finger domain-containing protein 1
AX-88225911	gene32671	ssa15	37426239	37459378	2.756823633	2.939303838	0.004529413		1	inverted formin-2-like%2C transcript variant X1
AX-88225911	gene30725	ssa14	41350313	41411417	15.91551382	2.939137235	0.004531563		1	nicotinamide riboside kinase 2-like%2C transcript variant X1
AX-88225911	gene8383	ssa03	89962845	89966991	10.60167877	2.9335191	0.004604604		1	uncharacterized LOC106602018
AX-88225911	gene45637	ssa23	29487954	29529404	4.965433464	2.933374857	0.004606493		1	long-chain-fatty-acid--CoA ligase ACSBG2-like%2C transcript variant X1
AX-88225911	gene79770	jcf1001176670_0_0	825	13453	1.595339154	2.932301106	0.004620582		1	E3 ubiquitin-protein ligase Mdm2-like
AX-88225911	gene6184	ssa03	13302155	13309027	17.64975691	2.931731714	0.004628069		1	calponin-2-like
AX-88225911	gene68599	jcf1000703746_0_0	6683	9930	693.0158783	2.930626665	0.004642632		1	barrier-to-autointegration factor-like
AX-88225911	gene20348	ssa09	136860531	136895025	6.613316119	2.929192261	0.004661598		1	transforming growth factor beta-1-like%2C transcript variant X1
AX-88225911	gene41323	ssa20	22287496	22323813	2.037107077	2.927113496	0.004689212		1	GTPase-activating protein and VPS9 domain-containing protein 1-like%2C transcript variant X1
AX-88225911	gene24247	ssa11	74193593	74215214	2.69008567	2.920784435	0.004774222		1	mothers against decapentaplegic homolog 6-like
AX-88225911	gene14723	ssa06	62306039	62348819	5.184247256	2.920015163	0.004784651		1	estrogen-related receptor gamma-like%2C transcript variant X1
AX-88225911	gene81373	jcf1001247548_0_0	2576	9119	3.95840914	2.919136256	0.004796592		1	lysosomal Pro-X carboxypeptidase-like
AX-88225911	gene31854	ssa14	89041258	89047434	1.542002967	2.91572462	0.004843207		1	peptidyl-prolyl cis-trans isomerase FKBP14
AX-88225911	gene8999	ssa04	15997310	15999235	1.830622236	2.914402543	0.004861383		1	transmembrane protein 109-like
AX-88225911	gene9867	ssa04	44515924	44518608	-2.77573025	-2.91326521	0.00487707		1	uncharacterized LOC106603408
AX-88225911	gene37340	ssa17	43633108	43649913	-0.76262254	-2.91279608	0.004883554		1	serine/threonine-protein phosphatase 6 regulatory subunit 2-like%2C transcript variant X1
AX-88225911	gene58225	jcf1000257751_0_0	6	13340	1.855738169	2.912304617	0.004890356		1	dynamin-2-like
AX-88225911	gene28334	ssa13	40457357	40470306	20.81401855	2.910197189	0.00491962		1	Ubiquitin-conjugating enzyme E2 variant 1
AX-88225911	gene35633	ssa16	72180456	72198429	-1.512067	-2.91006373	0.004921479		1	single-stranded DNA-binding protein 3-like%2C transcript variant X1
AX-88225911	gene37161	ssa17	36434211	36442666	4.035836428	2.908497859	0.004943336		1	aldose reductase-like%2C transcript variant X1
AX-88225911	gene79624	jcf1001172642_0_0	1714	7900	10.32983637	2.905999277	0.004978398		1	Decorin
AX-88225911	gene23985	ssa11	61539883	61545271	6.708808722	2.903424679	0.005014767		1	small nuclear ribonucleoprotein Sm D3
AX-88225911	gene33155	ssa15	63358567	63374840	3.428365756	2.902931195	0.005021766		1	interferon-related developmental regulator 2-like%2C transcript variant X1
AX-88225911	gene703	ssa01	29219774	29225435	15.2275123	2.897474801	0.005099757		1	ornithine decarboxylase 1
AX-88225911	gene44593	ssa22	37808885	37820655	15.38843271	2.896513809	0.005113609		1	caveolin 3
AX-88225911	gene7390	ssa03	60287926	60293602	7.662525405	2.896047121	0.005120348		1	DEXH (Asp-Glu-X-His) box polypeptide 58
AX-88225911	gene35431	ssa16	64447882	64461741	17.6321261	2.895706455	0.005125272		1	signal transducer and activator of transcription 1 alpha
AX-88225911	gene55025	jcf1000121779_0_0	3383	5050	1.255996733	2.894349287	0.005144935		1	methyltransferase WBSCR22
AX-88225911	gene11275	ssa05	29622529	29633955	13.80888602	2.893938689	0.005150897		1	insulin-like growth factor binding protein-like 1
AX-88225911	gene25909	ssa12	34898126	34980877	1.937228343	2.893485067	0.005157492		1	dystroglycan-like%2C transcript variant X1
AX-88225911	gene4566	ssa02	42598795	42605301	8.581749652	2.891367148	0.005188385		1	Importin subunit alpha-2
AX-88225911	gene26721	ssa12	70647884	70652399	1.886266224	2.891279827	0.005189662		1	snail homolog Sna
AX-88225911	gene46755	ssa24	24917643	24938024	-1.58194573	-2.89108614	0.005192496		1	Probable palmitoyltransferase ZDHHC12
AX-88225911	gene5998	ssa03	8051253	8127044	1.790343544	2.885274946	0.005278204		1	abl interactor 1-like
AX-88225911	gene17639	ssa09	9366380	9375411	4.258085482	2.882085761	0.005325794		1	heterogeneous nuclear ribonucleoprotein Q%2C transcript variant X1
AX-88225911	gene32796	ssa15	43555934	43561590	-0.91219461	-2.87762103	0.005393084		1	Cell growth regulator with RING finger domain protein 1
AX-88225911	gene405	ssa01	20391301	20418531	16.06620885	2.877345029	0.00539727		1	chloride intracellular channel 4
AX-88225911	gene25694	ssa12	24886989	24913639	11.76309699	2.877194099	0.00539956		1	uncharacterized LOC106564892
AX-88225911	gene27326	ssa12	91575598	91603870	1.693239492	2.876610403	0.005408424		1	pre-mRNA-splicing factor ISY1 homolog
AX-88225911	gene24973	ssa12	3274417	3278382	14.43942523	2.873142947	0.005461363		1	vascular cell adhesion protein 1-like
AX-88225911	gene23120	ssa11	22394430	22432649	-2.58350673	-2.86906543	0.005524228		1	dual specificity protein phosphatase 22-A-like%2C transcript variant X1
AX-88225911	gene19134	ssa09	78029547	78088976	2.239273363	2.868059933	0.005539833		1	glypican-4-like%2C transcript variant X1
AX-88225911	gene17820	ssa09	19183959	19190913	14.28106298	2.864973446	0.005587987		1	uncharacterized transmembrane protein DDB_G0289901-like
AX-88225911	gene3755	ssa02	13058265	13064049	85.96876558	2.864876226	0.00558951		1	urokinase plasminogen activator surface receptor-like

AX-88225911	gene14131	ssa06	38120783	38126472	9.482625076	2.8637307	0.005607485	1	2%2C3-cyclic-nucleotide 3-phosphodiesterase
AX-88225911	gene1914	ssa01	102550917	102557543	2.504092392	2.861313866	0.005645583	1	receptor-type tyrosine-protein phosphatase epsilon-like
AX-88225911	gene31432	ssa14	69022328	69036173	9.968154593	2.860500231	0.005658462	1	asparagine synthetase [glutamine-hydrolyzing]-like
AX-88225911	gene19921	ssa09	113687181	113693282	3.160165346	2.859872193	0.005668422	1	ras-related protein Rab-34-like%2C transcript variant X1
AX-88225911	gene354	ssa01	17678099	17704723	2.210365468	2.858880581	0.005684181	1	filamin-A-interacting protein 1-like%2C transcript variant X1
AX-88225911	gene6618	ssa03	33858715	33862955	27.18163473	2.857894502	0.005699893	1	guanine nucleotide-binding protein G(I)/G(S)/G(O) subunit gamma-5%2C transcript variant X1
AX-88225911	gene41430	ssa20	26318365	26326733	11.5980901	2.852505831	0.00578646	1	bifunctional methylenetetrahydrofolate dehydrogenase/cyclohydrolase%2C mitochondrial-like
AX-88225911	gene36483	ssa17	14690009	14706843	8.582671117	2.85215788	0.005792091	1	lipid phosphate phosphohydrolase 2-like%2C transcript variant X1
AX-88225911	gene32015	ssa15	3413295	3417252	24.4992023	2.850159315	0.005824533	1	ubiquitin-conjugating enzyme E2 L3-like
AX-88225911	gene13863	ssa06	31233349	31244616	43.3335052	2.849799344	0.005830394	1	CD9 antigen-like
AX-88225911	gene21674	ssa10	67615993	67694208	6.116141213	2.847214652	0.005872639	1	MOB kinase activator 2-like%2C transcript variant X1
AX-88225911	gene20177	ssa09	127221280	127226887	4.818066465	2.846102549	0.005890902	1	integral membrane protein 2C-like
AX-88225911	gene7644	ssa03	66652851	66665135	-0.67439537	-2.84581349	0.005895657	1	Histone acetyltransferase MYST2
AX-88225911	gene4138	ssa02	25186292	25191769	14.29583125	2.844811488	0.005912169	1	deoxyribonuclease-2-alpha-like%2C transcript variant X1
AX-88225911	gene6970	ssa03	48384858	48420925	0.948880775	2.844092668	0.005924041	1	BTB/POZ domain-containing adapter for CUL3-mediated RhoA degradation protein 3-like%2C transcript variant X1
AX-88225911	gene3144	ssa01	156442035	156453451	28.47942055	2.844080526	0.005924242	1	cystatin-1-like%2C transcript variant X1
AX-88225911	gene7383	ssa03	60008099	60013831	15.43487646	2.843847371	0.005928097	1	2'%2C3'-cyclic-nucleotide 3'-phosphodiesterase-like%2C transcript variant X1
AX-88225911	gene3797	ssa02	14151624	14163631	6.322955717	2.842633449	0.005948208	1	potassium-transporting ATPase alpha chain 1
AX-88225911	gene50337	ssa27	23396036	23478915	1.124254166	2.841943376	0.005959669	1	cytoplasmic linker associated protein 2%2C transcript variant X1
AX-88225911	gene15216	ssa07	172683	188535	1.999264673	2.841345324	0.005969617	1	eukaryotic translation initiation factor 4E type 2-like
AX-88225911	gene27868	ssa13	22389813	22404592	-0.88571531	-2.84109625	0.005973765	1	uncharacterized protein C6orf106 homolog
AX-88225911	gene330	ssa01	16599054	16606157	-1.9926162	-2.84022691	0.005988263	1	uncharacterized LOC106571589
AX-88225911	gene41378	ssa20	23913901	23934047	1.480219773	2.839440454	0.006001407	1	glutamine-fructose-6-phosphate transaminase 1
AX-88225911	gene43318	ssa21	31253780	31258747	2.222027514	2.838352069	0.006019641	1	uncharacterized protein C3orf38-like%2C transcript variant X1
AX-88225911	gene38851	ssa18	55753718	55764224	2.56977024	2.837234388	0.006038419	1	interferon-induced very large GTPase 1-like%2C transcript variant X1
AX-88225911	gene33059	ssa15	59230109	59304740	0.933652928	2.835736121	0.006063675	1	protein bicaudal D homolog 2-like%2C transcript variant X1
AX-88225911	gene32395	ssa15	25791617	25813642	13.60875238	2.834978729	0.00607648	1	14-3-3 protein zeta%2C transcript variant X1
AX-88225911	gene43843	ssa22	6176492	6183688	-2.0930194	-2.83457461	0.006083322	1	protein kish-B-like
AX-88225911	gene29579	ssa13	100120889	100311797	1.995013402	2.833737293	0.006097521	1	Rho GTPase activating protein 24%2C transcript variant X1
AX-88225911	gene30572	ssa14	35561234	35564993	-2.89688623	-2.83207255	0.006125843	1	abhydrolase domain-containing protein 4-like%2C transcript variant X1
AX-88225911	gene31201	ssa14	59522300	59526637	77.29587138	2.831336916	0.006138396	1	VHSV-induced protein-like
AX-88225911	gene20535	ssa10	10965048	10973624	20.08961426	2.830854458	0.006146643	1	galectin-3-binding protein A-like%2C transcript variant X1
AX-88225911	gene39129	ssa18	63955193	63959790	4.475641073	2.829134261	0.006176127	1	purine nucleoside phosphorylase-like
AX-88225911	gene447	ssa01	22036342	22039388	2.140164057	2.827492004	0.006204396	1	mitotic checkpoint serine/threonine-protein kinase BUB1 beta-like
AX-88225911	gene33152	ssa15	63202819	63208478	3.817458443	2.827221473	0.006209065	1	semaphorin-3F-like
AX-88225911	gene24596	ssa11	83195484	83211679	1.642582346	2.826939045	0.006213942	1	myozenin-2-like%2C transcript variant X1
AX-88225911	gene961	ssa01	42469070	42474127	46.540526	2.826324268	0.00622457	1	Cathepsin B
AX-88225911	gene14668	ssa06	60255115	60280335	1.622068248	2.825545931	0.00623805	1	uridine-cytidine kinase 1-like 1
AX-88225911	gene24441	ssa11	78362795	78377849	49.34242673	2.824422185	0.00625756	1	lipocalin-like%2C transcript variant X1
AX-88225911	gene59872	jcf1000333282_0_0	80	5185	1.768332786	2.822618575	0.006288991	1	nectin-4-like%2C transcript variant X1
AX-88225911	gene19264	ssa09	84602516	84620695	1.75614913	2.819395148	0.006345526	1	coiled-coil domain containing 69%2C transcript variant X1
AX-88225911	gene68563	jcf1000702769_0_0	899	1941	3.098203513	2.819159707	0.006349673	1	B9 protein domain 1
AX-88225911	gene10045	ssa04	49559124	49668754	0.720260955	2.815403409	0.006416184	1	coxsackievirus and adenovirus receptor homolog
AX-88225911	gene42992	ssa21	16454531	16458214	4.415685825	2.814435698	0.006433422	1	cytochrome b reductase 1
AX-88225911	gene17522	ssa08	26405404	26421466	17.39602682	2.813825415	0.006444315	1	thioredoxin-like%2C transcript variant X1

AX-88225911	gene36800	ssa17	23641825	23647362	3.486696188	2.812713462	0.006464207	1	importin-5-like
AX-88225911	gene29121	ssa13	80950617	80976276	27.35931826	2.810379294	0.006506146	1	moesin%2C transcript variant X1
AX-88225911	gene36660	ssa17	19104025	19116410	10.53319975	2.809279489	0.006525993	1	calcipressin-1-like%2C transcript variant X1
AX-88225911	gene20861	ssa10	22003394	22043995	-1.10769974	-2.80337536	0.006633495	1	uncharacterized protein C1orf21 homolog%2C transcript variant X1
AX-88225911	gene1209	ssa01	56533305	56587353	2.19973355	2.802257506	0.006654031	1	V-type proton ATPase subunit B%2C brain isoform
AX-88225911	gene22695	ssa10	115536080	115553190	53.25982244	2.802192428	0.006655229	1	annexin A2-like
AX-88225911	gene3430	ssa02	6265950	6269953	7.488577623	2.801213774	0.006673259	1	protein asteroid homolog 1-like
AX-88225911	gene53862	jcf1000071781_0-0_0_0	705	2263	5.025160185	2.801055065	0.006676187	1	dynamin-2-like
AX-88225911	gene47413	ssa25	12713736	12755891	3.128871426	2.799841259	0.006698621	1	RNA-binding motif%2C single-stranded-interacting protein 1-like%2C transcript variant X1
AX-88225911	gene24402	ssa11	77701603	77786972	1.963206533	2.798151127	0.006729973	1	tyrosine-protein kinase receptor UFO-like%2C transcript variant X1
AX-88225911	gene15943	ssa07	26163903	26189894	17.33039652	2.79779549	0.006736588	1	decorin
AX-88225911	gene28163	ssa13	35103280	35152144	1.108156132	2.796578531	0.006759267	1	formin-like protein 3%2C transcript variant X1
AX-88225911	gene14935	ssa06	72678390	72692064	1.365981331	2.795811555	0.006773596	1	heterogeneous nuclear ribonucleoprotein U-like
AX-88225911	gene2317	ssa01	119592361	119609431	2.42826281	2.795643571	0.006776738	1	ankyrin repeat and LEM domain-containing protein 2-like
AX-88225911	gene10657	ssa05	611711	665090	8.502625977	2.7956243	0.006777098	1	plastin-3-like
AX-88225911	gene24493	ssa11	79565867	79569834	2.926372762	2.794246874	0.006802915	1	NEDD4-binding protein 2-like 1
AX-88225911	gene73365	jcf1000908453_0_0	2384	6112	3.924468659	2.793200782	0.006822582	1	uncharacterized LOC106595094
AX-88225911	gene15875	ssa07	24189776	24252760	3.112299058	2.791240274	0.006859581	1	Astrocytic phosphoprotein PEA-15
AX-88225911	gene43083	ssa21	21070602	21137050	2.910146621	2.788185205	0.006917604	1	collagen alpha-2(V) chain-like
AX-88225911	gene2291	ssa01	118407950	118417300	3.846324295	2.786181476	0.006955904	1	leukemia inhibitory factor receptor-like%2C transcript variant X1
AX-88225911	gene4859	ssa02	52749244	52750574	-1.16790965	-2.78583035	0.006962636	1	LisH domain-containing protein C16orf63 homolog
AX-88225911	gene19311	ssa09	88712148	88742308	8.745652586	2.78405729	0.006996719	1	glutathione peroxidase 3-like
AX-88225911	gene6532	ssa03	29465773	29468022	3.112391276	2.780519847	0.007065178	1	forkhead box C1-A
AX-88225911	gene12116	ssa05	60823150	60839401	1.674045371	2.77917349	0.007091395	1	valine--tRNA ligase-like%2C transcript variant X1
AX-88225911	gene12489	ssa05	70690050	70698512	8.525707541	2.777758381	0.007119047	1	CD209 antigen-like protein E%2C transcript variant X1
AX-88225911	gene24448	ssa11	78482543	78490388	15.96126922	2.773985292	0.007193259	1	uncharacterized LOC106563597
AX-88225911	gene11057	ssa05	16299367	16304042	260.6902048	2.770087455	0.00727067	1	thymosin beta
AX-88225911	gene41675	ssa20	37282580	37298573	-0.70070598	-2.76939456	0.007284511	1	excision repair cross-complementation group 8%2C transcript variant X1
AX-88225911	gene35364	ssa16	61003278	61049711	7.155404834	2.767491559	0.007322648	1	amyloid beta A4 protein-like
AX-88225911	gene48054	ssa25	42204605	42268085	5.177817152	2.764983976	0.007373181	1	collagen alpha-2(V) chain-like
AX-88225911	gene13457	ssa06	22170260	22174194	5.870497217	2.764663809	0.007379656	1	uncharacterized LOC106606813
AX-88225911	gene40531	ssa19	63391614	63418578	5.589279363	2.764102244	0.007391026	1	interferon regulatory factor 3
AX-88225911	gene1767	ssa01	94086501	94101640	11.80831443	2.763608836	0.007401029	1	serglycin-like
AX-88225911	gene25064	ssa12	6202276	6224916	1.659636997	2.763031532	0.007412749	1	trichohyalin-like%2C transcript variant X1
AX-88225911	gene27704	ssa13	16614388	16626167	-1.60729518	-2.76048241	0.007464701	1	ankyrin repeat and BTB (POZ) domain containing 1%2C transcript variant X1
AX-88225911	gene41221	ssa20	18336763	18388054	1.120676314	2.759751056	0.007479668	1	tumor necrosis factor ligand superfamily member 6-like
AX-88225911	gene47759	ssa25	28809463	28823873	9.031909323	2.759587223	0.007483024	1	epithelial stromal interaction 1 (breast)%2C transcript variant X1
AX-88225911	gene4509	ssa02	40960693	40961702	124.6072405	2.75905507	0.007493937	1	14 kDa transmembrane protein
AX-88225911	gene41405	ssa20	25704232	25708484	5.889221107	2.758177942	0.007511954	1	L-serine dehydratase/L-threonine deaminase-like
AX-88225911	gene22169	ssa10	92802577	92817230	-0.41573536	-2.75776797	0.00752039	1	uncharacterized LOC106561283%2C transcript variant X1
AX-88225911	gene51787	ssa29	16633838	16655009	0.898548797	2.755853313	0.007559899	1	notchless homolog 1 (Drosophila)%2C transcript variant X1
AX-88225911	gene25103	ssa12	7216571	7228195	40.76635244	2.755616296	0.007564803	1	GTPase IMAP family member 7-like
AX-88225911	gene41524	ssa20	29203093	29206653	1.262198908	2.755096231	0.007575574	1	DIM1 dimethyladenosine transferase 1 homolog
AX-88225911	gene80009	jcf1001187899_0_0	4152	5351	-3.87108714	-2.75358621	0.007606927	1	uncharacterized LOC106597250
AX-88225911	gene16991	ssa08	8321602	9674890	-0.69120526	-2.75051528	0.007671058	1	zinc finger protein 329-like%2C transcript variant X1
AX-88225911	gene24254	ssa11	74380824	74407954	1.877632426	2.749111426	0.007700539	1	anoctamin-5-like%2C transcript variant X1
AX-88225911	gene51465	ssa28	33012291	33049053	2.230600854	2.748921118	0.007704544	1	ribosome-binding protein 1-like%2C transcript variant X1

AX-88225911	gene6217	ssa03	14459474	14466546	-2.02852652	-2.74777866	0.007728624		1	choline-phosphate cytidylyltransferase A-like%2C transcript variant X1
AX-88225911	gene3796	ssa02	14126006	14140527	73.30467812	2.747653971	0.007731256		1	annexin A7-like
AX-88225911	gene13440	ssa06	21329779	21344974	2.497319686	2.746299709	0.007759899		1	Eukaryotic peptide chain release factor GTP-binding subunit ERF3B
AX-88225911	gene37861	ssa18	6061856	6068368	11.02341656	2.745776345	0.007770994		1	BCL2-associated athanogene 3
AX-88225911	gene29415	ssa13	92422467	92427021	2.015965267	2.745295324	0.007781205		1	peptidylprolyl isomerase domain and WD repeat containing 1
AX-88225911	gene2196	ssa01	115462617	115467939	6.755719352	2.74388359	0.007811242		1	ubiquitin-conjugating enzyme E2 L3-like
AX-88225911	gene24470	ssa11	78857426	78869567	1.353446975	2.742515146	0.007840459		1	UDP-glucose 6-dehydrogenase-like
AX-88225911	gene915	ssa01	41142173	41151803	-2.67537973	-2.74168455	0.007858242		1	microtubule-associated protein RP/EB family member 3-like%2C transcript variant X1
AX-88225911	gene18813	ssa09	65568701	65579911	3.835673387	2.737993766	0.007937708		1	Vacuolar proton translocating ATPase 116 kDa subunit a
AX-88225911	gene30253	ssa14	23846457	23851700	-1.10193134	-2.73693946	0.007960543		1	transmembrane protein 68-like%2C transcript variant X1
AX-88225911	gene35089	ssa16	44797859	44810537	1.168375469	2.735529837	0.007991168		1	RNA 3'-terminal phosphate cyclase%2C transcript variant X1
AX-88225911	gene41237	ssa20	18701188	18722636	-1.67193114	-2.73459004	0.008011645		1	ureidopropionase%2C beta
AX-88225911	gene5364	ssa02	66114094	66132002	1.854768378	2.734296508	0.008018051		1	dynamin-2-like
AX-88225911	gene39566	ssa19	12844897	12869198	3.488328898	2.734280911	0.008018391		1	SH3-domain GRB2-like endophilin B1
AX-88225911	gene50648	ssa27	38111889	38114220	9.301933759	2.733423789	0.008037124		1	MARCKS-related protein
AX-88225911	gene7342	ssa03	59029283	59075678	6.504024435	2.732007484	0.008068165		1	LIM and SH3 domain protein 1-like
AX-88225911	gene28336	ssa13	40482787	40484397	15.36130898	2.731521494	0.008078841		1	CCAAT/enhancer binding protein (C/EBP)%2C beta
AX-88225911	gene38092	ssa18	16043637	16080659	2.240176578	2.729299127	0.008127829		1	serine/threonine-protein kinase VRK1-like%2C transcript variant X1
AX-88225911	gene44333	ssa22	28285177	28335712	1.158426239	2.72824403	0.008151182		1	rho guanine nucleotide exchange factor 25-like%2C transcript variant X1
AX-88225911	gene9848	ssa04	43777479	43810316	30.60865152	2.727887316	0.008159091		1	Moesin
AX-88225911	gene33382	ssa15	72244905	72248458	-1.11543007	-2.72743644	0.008169098		1	MRG-binding protein
AX-88225911	gene36036	ssa16	83311860	83317130	2.79418918	2.724889341	0.008225841		1	Autophagy-related protein 3
AX-88225911	gene38762	ssa18	53541267	53555751	34.24091626	2.724454808	0.008235557		1	placenta-specific gene 8 protein-like%2C transcript variant X1
AX-88225911	gene23495	ssa11	37597839	37624167	-1.38534595	-2.72308968	0.00826615		1	ATP-dependent Clp protease ATP-binding subunit clpX-like%2C mitochondrial
AX-88225911	gene7045	ssa03	50256938	50270883	1.517895342	2.720030748	0.008335079		1	U1 small nuclear ribonucleoprotein 70 kDa-like
AX-88225911	gene21104	ssa10	31684506	31735693	22.85300479	2.719978433	0.008336263		1	proteoglycan 4-like
AX-88225911	gene11851	ssa05	54116801	54120854	16.63822067	2.719749962	0.008341433		1	protein C-ets-2-like%2C transcript variant X1
AX-88225911	gene9967	ssa04	47118194	47128239	1.324299582	2.719379574	0.00834982		1	protein sprouty homolog 3-like%2C transcript variant X1
AX-88225911	gene22979	ssa11	18123221	18140388	3.98222913	2.719330553	0.008350931		1	eukaryotic translation initiation factor 4 gamma 2-like
AX-88225911	gene6340	ssa03	19489495	19501508	12.2963897	2.71754069	0.008391578		1	cAMP-responsive element modulator-like%2C transcript variant X1
AX-88225911	gene37625	ssa17	52268366	52279334	1.281437105	2.717424193	0.008394229		1	KRR1 small subunit processome component homolog
AX-88225911	gene48256	ssa26	2656057	2659926	3.581636614	2.715993249	0.008426865		1	serine/threonine-protein phosphatase 2A catalytic subunit beta isoform-like
AX-88225911	gene23040	ssa11	20793071	20892019	-0.44595959	-2.71496063	0.008450487		1	zinc finger protein 280D%2C transcript variant X1
AX-88225911	gene29859	ssa14	10336066	10354867	4.555974891	2.712117036	0.008515852		1	V-type proton ATPase subunit d 1-like
AX-88225911	gene13623	ssa06	24334596	24338512	-0.88114509	-2.71088217	0.008544382		1	transmembrane and ubiquitin-like domain containing 2
AX-88225911	gene19751	ssa09	108111740	108116189	2.462769109	2.709986707	0.008565124		1	Cyclic AMP-dependent transcription factor ATF-5
AX-88225911	gene48815	ssa26	29496981	29499693	-2.28386632	-2.70814265	0.008607985		1	Membrane-spanning 4-domains subfamily A member 4A
AX-88225911	gene44992	ssa22	56980910	57103193	1.343922824	2.707980354	0.008611767		1	semaphorin-3D-like
AX-88225911	gene19348	ssa09	90838275	90851386	2.262111943	2.707055154	0.008633354		1	#N/A
AX-88225911	gene1474	ssa01	74396708	74403348	5.311101101	2.7055948	0.008667527		1	28 kDa heat- and acid-stable phosphoprotein-like%2C transcript variant X1
AX-88225911	gene44053	ssa22	15757194	15761184	-1.21169827	-2.7015487	0.008762856		1	bcl-2-like protein 1
AX-88225911	gene13819	ssa06	29993781	29995614	7.731478525	2.699236276	0.008817768		1	transcription factor jun-B-like
AX-88225911	gene23436	ssa11	35800426	35811384	1.162972237	2.699005685	0.008823261		1	reticulocalbin 1%2C EF-hand calcium binding domain
AX-88225911	gene26011	ssa12	41601932	41619861	7.046700672	2.698576675	0.008833489		1	protein-glutamine gamma-glutamyltransferase 2-like
AX-88225911	gene41199	ssa20	17602874	17619544	3.402469954	2.698087083	0.008845174		1	fibroblast growth factor 17-like%2C transcript variant X1
AX-88225911	gene6722	ssa03	36416602	36435790	8.954561583	2.697778635	0.008852543		1	SGT1%2C suppressor of G2 allele of SKP1 (S. cerevisiae)%2C transcript variant X1

AX-88225911	gene52449	jcf1000008525_0_0	4574	7517	24.52122716	2.697589927	0.008857055	1	extracellular matrix protein 1-like
AX-88225911	gene14846	ssa06	67875621	67893679	1.707659856	2.696998512	0.008871206	1	poly (ADP-ribose) polymerase 1%2C transcript variant X1
AX-88225911	gene20228	ssa09	130523380	130537958	1.871415328	2.696950224	0.008872363	1	arf-GAP with Rho-GAP domain%2C ANK repeat and PH domain-containing protein 1-like
AX-88225911	gene36797	ssa17	23600165	23603709	9.100900146	2.693570651	0.008953638	1	uncharacterized LOC106575706
AX-88225911	gene23933	ssa11	58197665	58213379	14.14715278	2.692639474	0.008976151	1	V-type proton ATPase subunit G 1-like
AX-88225911	gene38172	ssa18	20617475	20620314	-3.47539315	-2.69201672	0.008991236	1	vitamin K epoxide reductase complex subunit 1-like protein 1
AX-88225911	gene40338	ssa19	54843734	54854948	57.26036461	2.69108819	0.009013771	1	V-type proton ATPase 16 kDa proteolipid subunit
AX-88225911	gene47590	ssa25	20767006	20774150	3.046597426	2.690986767	0.009016236	1	dnaJ homolog subfamily B member 11-like
AX-88225911	gene32554	ssa15	33066152	33068861	4.499740316	2.690727034	0.00902255	1	podocan-like
AX-88225911	gene42783	ssa21	1043557	1055205	4.847242871	2.690365504	0.009031346	1	COMM domain containing 6
AX-88225911	gene17709	ssa09	14085888	14145504	1.037866432	2.68925624	0.009058383	1	uncharacterized LOC106610761%2C transcript variant X1
AX-88225911	gene48513	ssa26	17733127	17753326	45.6786212	2.687201934	0.00910865	1	pyruvate kinase PKM-like%2C transcript variant X1
AX-88225911	gene47453	ssa25	14259104	14261577	1.464067028	2.685720712	0.009145052	1	cytochrome b reductase 1-like
AX-88225911	gene23845	ssa11	55221721	55224993	4.504134767	2.683051448	0.009210986	1	tubulin alpha chain%2C testis-specific-like
AX-88225911	gene10449	ssa04	74125077	74138110	5.982695914	2.680496486	0.009274503	1	lymphocyte cytosolic protein 2-like%2C transcript variant X1
AX-88225911	gene25785	ssa12	27978572	28098722	1.087686663	2.678494259	0.009324557	1	signal-induced proliferation-associated 1-like protein 2%2C transcript variant X1
AX-88225911	gene41749	ssa20	38010053	38011202	8.087613572	2.675719269	0.009394339	1	isotocin-neurophysin IT 2-like
AX-88225911	gene6308	ssa03	17527365	17535070	6.084196631	2.675605907	0.009397199	1	integral membrane protein 2C-like
AX-88225911	gene39111	ssa18	63336179	63346571	3.070408225	2.674258364	0.009431266	1	heterogeneous nuclear ribonucleoprotein C-like%2C transcript variant X1
AX-88225911	gene26307	ssa12	52736859	52752507	3.315427474	2.671958356	0.009489673	1	arginyl aminopeptidase (aminopeptidase B)
AX-88225911	gene29023	ssa13	78065660	78071336	-2.58483166	-2.67177693	0.009494294	1	E3 ubiquitin-protein ligase RNF167-like%2C transcript variant X1
AX-88225911	gene33658	ssa15	83368975	83383443	5.750446475	2.671667416	0.009497084	1	general receptor for phosphoinositides 1-associated scaffold protein-like
AX-88225911	gene30498	ssa14	33737828	33752397	-0.89297518	-2.67145354	0.009502536	1	Vasculin-like protein 1
AX-88225911	gene40487	ssa19	60275356	60292415	3.532810921	2.669096896	0.009562797	1	transcription elongation factor B (SIII)%2C polypeptide 2 (18kDa%2C elongin B)%2C transcript variant X1
AX-88225911	gene1059	ssa01	48925721	48956074	1.982277838	2.668983785	0.009565698	1	phosphoinositide 3-kinase adapter protein 1-like%2C transcript variant X1
AX-88225911	gene3023	ssa01	149796152	149819776	2.669450638	2.668319832	0.009582743	1	interleukin-6 receptor subunit beta-like
AX-88225911	gene16294	ssa07	42417658	42430371	3.201112175	2.666907364	0.009619097	1	V-type proton ATPase subunit E 1-like
AX-88225911	gene52012	ssa29	26569909	26588258	5.655250606	2.665875191	0.009645742	1	V-type proton ATPase catalytic subunit A
AX-88225911	gene37193	ssa17	37594963	37613759	4.667585579	2.664931175	0.00967017	1	nicotinamide phosphoribosyltransferase
AX-88225911	gene51682	ssa29	11930960	11997272	1.097244296	2.662997295	0.00972039	1	collectin-12-like
AX-88225911	gene19103	ssa09	77358844	77368328	1.767652504	2.662958761	0.009721393	1	EH domain-containing protein 1-like%2C transcript variant X1
AX-88225911	gene48523	ssa26	18370001	18372562	9.901674042	2.662470356	0.009734114	1	ADM-like
AX-88225911	gene32364	ssa15	24154097	24213019	12.54235724	2.662274927	0.009739209	1	ezrin
AX-88225911	gene9250	ssa04	25664922	25734833	12.55600374	2.661665421	0.009755114	1	CD166 antigen homolog
AX-88225911	gene50596	ssa27	35575492	35582561	3.503997481	2.661197061	0.009767352	1	RNA-binding protein 43-like%2C transcript variant X1
AX-88225911	gene35055	ssa16	43549789	43558483	9.563101592	2.660649545	0.009781676	1	interferon-induced protein 44-like%2C transcript variant X1
AX-88225911	gene23975	ssa11	61201038	61222725	1.407576027	2.659234974	0.009818772	1	ER degradation-enhancing alpha-mannosidase-like protein 3%2C transcript variant X1
AX-88225911	gene33272	ssa15	68040802	68043031	3.372654742	2.65811878	0.009848134	1	tumor necrosis factor receptor superfamily member 6B-like
AX-88225911	gene51716	ssa29	13197488	13268738	0.800768962	2.657948714	0.009852614	1	arf-GAP with SH3 domain%2C ANK repeat and PH domain-containing protein 1-like%2C transcript variant X1
AX-88225911	gene40662	ssa19	68799549	68817158	5.590104454	2.656110463	0.009901165	1	interferon regulatory factor 9
AX-88225911	gene28191	ssa13	35875239	35889708	5.085482973	2.656011386	0.009903788	1	LIM domain and actin-binding protein 1-like%2C transcript variant X1
AX-88225911	gene5773	ssa03	6293359	6305698	8.474255976	2.652962722	0.009984806	1	ATP-binding cassette%2C sub-family F (GCN20)%2C member 2
SNP	gene	gene_chr	gene_start	gene_end	beta	t-stat	p-value	FDR	Annotation
AX-98325250	gene69020	jcf1000721884_0_0	34279	43741	5.715728827	6.967589083	1.85E-09	0.008497461	stonustoxin subunit beta-like
AX-98325250	gene7474	ssa03	62954778	62969268	-4.261415117	-4.664019724	1.56E-05	1	SUMO-conjugating enzyme UBC9-B%2C transcript variant X1

AX-98325250	gene13192	ssa06	13143954	13160089	-2.555025796	-4.624509688	1.80E-05	1	lipopolysaccharide-induced tumor necrosis factor-alpha factor homolog%2C transcript variant X1
AX-98325250	gene48192	ssa25	50735003	50738314	2.600462961	3.807010702	0.000310652	1	SMT3 suppressor of mif two 3 homolog 3 (S. cerevisiae)
AX-98325250	gene9255	ssa04	26314846	26376245	32.54083539	3.716041468	0.000418972	1	fibroin heavy chain-like%2C transcript variant X1
AX-98325250	gene24679	ssa11	86227658	86281385	7.369763531	3.608303551	0.000594027	1	ubiquitin-like 3
AX-98325250	gene6982	ssa03	48841655	48842306	4.474508493	3.520720109	0.000785638	1	ferritin%2C middle subunit-like
AX-98325250	gene8514	ssa04	1591153	1596870	3.913163305	3.512195532	0.000807142	1	UPF0462 protein C4orf33 homolog
AX-98325250	gene38394	ssa18	32555367	32557659	7.480850358	3.448341166	0.000986916	1	probable lysosomal cobalamin transporter
AX-98325250	gene15522	ssa07	12101875	12122500	0.914992385	3.445949566	0.000994336	1	phospholipid scramblase 2-like%2C transcript variant X1
AX-98325250	gene7632	ssa03	66390418	66421393	1.837847841	3.370982308	0.001255572	1	rho GTPase-activating protein 12-like%2C transcript variant X1
AX-98325250	gene47646	ssa25	22411993	22418123	1.867941191	3.357511436	0.001308906	1	disrupted in renal carcinoma 2
AX-98325250	gene67706	jcf1000669051_0_0	33867	46196	3.821609206	3.312692265	0.001502157	1	uncharacterized LOC106591321
AX-98325250	gene42022	ssa20	49992608	50020170	-0.957061524	-3.259410408	0.001766847	1	Hypoxia-inducible factor 1 alpha
AX-98325250	gene6542	ssa03	30221745	30224060	0.509271986	3.244927881	0.001846037	1	zinc finger protein 830-like
AX-98325250	gene56456	jcf1000186705_0_0	10562	11029	10.91213227	3.238481865	0.001882346	1	#N/A
AX-98325250	gene18836	ssa09	66457793	66489558	-0.404364806	-3.12276836	0.002659711	1	F-box/WD repeat-containing protein 11%2C transcript variant X1
AX-98325250	gene2702	ssa01	136162651	136168052	-0.553646806	-3.110092064	0.002761098	1	chromosome ssa01 open reading frame%2C human C2orf42%2C transcript variant X1
AX-98325250	gene50859	ssa28	5071285	5096360	2.234101199	3.100110731	0.00284346	1	Serine protease HTRA1
AX-98325250	gene62725	jcf1000450480_0_0	1213	6547	-1.607200982	-3.095799935	0.002879735	1	protein lifeguard 1-like
AX-98325250	gene12518	ssa05	71173230	71175948	26.0495987	3.052032255	0.003273305	1	uncharacterized LOC106605867
AX-98325250	gene35002	ssa16	42102742	42108138	-0.7300671	-3.045264663	0.003338458	1	large subunit GTPase 1 homolog
AX-98325250	gene23872	ssa11	55831721	55863763	2.626866661	3.036374551	0.003425881	1	hyaluronan and proteoglycan link protein 1%2C transcript variant X1
AX-98325250	gene31970	ssa15	1791467	1809166	7.68910649	3.034276902	0.003446817	1	3-hydroxy-3-methylglutaryl-CoA reductase
AX-98325250	gene23559	ssa11	40189618	40193625	-3.050918569	-3.004502931	0.003757118	1	ADP-ribosylation factor GTPase-activating protein 2-like
AX-98325250	gene51836	ssa29	19618279	19736439	1.510409297	2.994412127	0.003868044	1	microtubule-associated protein 4-like%2C transcript variant X1
AX-98325250	gene41058	ssa20	12923289	12927934	0.725864201	2.993611355	0.003876976	1	serologically defined colon cancer antigen 3 homolog%2C transcript variant X1
AX-98325250	gene25744	ssa12	26605877	26639062	1.542550341	2.990363359	0.003913401	1	sprouty-related%2C EVH1 domain-containing protein 2-like%2C transcript variant X1
AX-98325250	gene24770	ssa11	91021002	91235731	6.21833984	2.985566177	0.003967781	1	hephaestin-like protein 1
AX-98325250	gene21272	ssa10	40952295	40991009	46.25056065	2.985415687	0.003969498	1	epithelial chloride channel protein-like
AX-98325250	gene20675	ssa10	15718194	15727373	214.6970252	2.980926322	0.004021045	1	tRNA-Ala
AX-98325250	gene17316	ssa08	20195570	20197752	9.956725472	2.971452384	0.004131869	1	ladderlectin-like
AX-98325250	gene26265	ssa12	51711011	51753397	0.784889668	2.966805087	0.004187262	1	inner centromere protein-like
AX-98325250	gene8115	ssa03	80008956	80016128	1.088605745	2.959597829	0.004274533	1	ras-related C3 botulinum toxin substrate 1-like
AX-98325250	gene19017	ssa09	74649053	74662462	6.937600553	2.949554469	0.004398962	1	Fatty aldehyde dehydrogenase
AX-98325250	gene50615	ssa27	36802272	36836611	1.043585685	2.948354973	0.004414045	1	phosphatidylinositide phosphatase SAC1-B-like
AX-98325250	gene7188	ssa03	54047961	54059117	28.74180069	2.942363711	0.004490102	1	membrane-spanning 4-domains subfamily A member 12-like
AX-98325250	gene21942	ssa10	80843601	80847917	-1.851241705	-2.939621056	0.004525323	1	C-factor-like%2C transcript variant X1
AX-98325250	gene9379	ssa04	27021672	27042969	-1.498095906	-2.93807185	0.004545331	1	argininosuccinate lyase
AX-98325250	gene2671	ssa01	135076875	135105974	0.95546137	2.934729105	0.004588781	1	anaphase-promoting complex subunit CDC26-like%2C transcript variant X1
AX-98325250	gene39298	ssa18	69479494	69494194	-0.597406574	-2.920333958	0.004780326	1	SMAD family member 1%2C transcript variant X1
AX-98325250	gene31200	ssa14	59519479	59522216	-1.281329129	-2.906468848	0.004971791	1	vacuolar protein sorting-associated protein 52 homolog%2C transcript variant X1
AX-98325250	gene66269	jcf1000604957_0-0_0_0	618	2074	7.940928698	2.88373612	0.005301118	1	stonustoxin subunit beta-like
AX-98325250	gene71321	jcf1000823330_0_0	318	1492	13.16666218	2.883183957	0.005309362	1	Type-2 ice-structuring protein
AX-98325250	gene27580	ssa13	10329381	10356731	5.407432733	2.873978677	0.00544856	1	solute carrier family 6 (neurotransmitter transporter)%2C member 8
AX-98325250	gene57456	jcf1000227469_0_0	4537	9463	41.97014321	2.845437479	0.005901848	1	ladderlectin-like
AX-98325250	gene4973	ssa02	55478997	55485143	20.38001152	2.834167132	0.006090228	1	tetraspanin-1-like

AX-98325250	gene67048	jcf1000638784_0_0	8	754	1.648295468	2.833994149	0.006093162	1	desmoplakin-like
AX-98325250	gene62203	jcf1000430681_0_0	6	1700	-1.982447266	-2.816172621	0.006402512	1	alpha-actinin-2-like
AX-98325250	gene54416	jcf1000095331_0_0	3	1238	5.893677597	2.800199107	0.006692	1	uncharacterized LOC106600005
AX-98325250	gene8639	ssa04	4879867	4882378	13.27173274	2.782674018	0.007023416	1	CD209 antigen-like
AX-98325250	gene24262	ssa11	74575400	74601706	1.019688591	2.765289177	0.007367014	1	leucine-rich repeat-containing G-protein coupled receptor 4-like
AX-98325250	gene27325	ssa12	91463760	91517650	-0.590132599	-2.754562419	0.007586644	1	probable E3 ubiquitin-protein ligase makorin-2%2C transcript variant X1
AX-98325250	gene51192	ssa28	18694431	18707501	25.06634811	2.752246592	0.007634842	1	putative all-trans-retinol 13%2C14-reductase
AX-98325250	gene59453	jcf1000312156_0_0	3140	5550	-7.829373226	-2.728326318	0.008149358	1	#N/A
AX-98325250	gene3918	ssa02	17862001	17881682	-4.274296201	-2.726971706	0.008179424	1	pituitary tumor-transforming gene 1 protein-interacting protein-like
AX-98325250	gene3435	ssa02	6295055	6298926	32.94243359	2.726902003	0.008180974	1	ectonucleotide pyrophosphatase/phosphodiesterase family member 7-like
AX-98325250	gene2399	ssa01	121655779	121674542	3.524338776	2.723116437	0.008265549	1	sodium-dependent phosphate transporter 1-B-like
AX-98325250	gene74293	jcf1000945758_0_0	839	74187	1.864923705	2.716272665	0.008420483	1	oxysterol-binding protein-related protein 10-like
AX-98325250	gene66532	jcf1000616619_0_0	5453	11365	-1.391974556	-2.69982568	0.008803742	1	alpha-actinin-2-like
AX-98325250	gene9973	ssa04	47242528	47246618	1.295538643	2.699486095	0.008811821	1	RNA binding motif protein%2C X-linked 2
AX-98325250	gene42199	ssa20	56840060	56927698	1.621880983	2.697616292	0.008856424	1	elastin-like%2C transcript variant X1
AX-98325250	gene20012	ssa09	116726413	116740505	0.919136979	2.690717831	0.009022774	1	beta-secretase 2-like
AX-98325250	gene30992	ssa14	51905386	51920082	6.414890828	2.679722025	0.009293835	1	translation initiation factor IF-2-like
AX-98325250	gene21904	ssa10	79319344	79337287	-0.746432767	-2.674482184	0.0094256	1	Homocysteine-responsive endoplasmic reticulum-resident ubiquitin-like domain member 1 protein
AX-98325250	gene15552	ssa07	12896222	12911045	9.894881108	2.669850788	0.009543482	1	excitatory amino acid transporter 3-like%2C transcript variant X1
AX-98325250	gene13929	ssa06	32809461	32855753	0.7061186	2.660704753	0.009780231	1	rho GTPase-activating protein 27-like%2C transcript variant X1
AX-98325250	gene26324	ssa12	53202960	53217834	1.433070336	2.65361093	0.00996753	1	leucine-rich repeat flightless-interacting protein 2-like%2C transcript variant X1

***Supplemental table S2. Highlighted immune genes with differential expression in H- versus L-gEBV salmon associated with SNP allele at locus* AX-88225911 (Ssa03 position 89781579).** *Chromosome, start and end map base positions for each gene are shown.*

Gene	Function	Position	*p*-value
Transmembrane	Immune	ssa17:23600165-23603709	0.009
tissue factor-like	Immune Acute phase	ssa14:15422983-15426979	0.004
MHC class I antigen	Immune Antigen presentation	ssa27:10122007-10149393	0.004
class I histocompatibility antigen, F10 alpha chain-like	Immune Antigen presentation	ssa27:10648406-10658002	0.004
Phosphoinositide 3-kinase adapter protein 1	Immune B cell	ssa01:48925721-48956074	0.010
myeloid cell surface antigen CD33-like	Immune cell marker	ssa19:74288873-74346695	0.004
C-X-C motif chemokine 11-like	Immune Chemokine	ssa12:19630840-19632724	0.003
C-C motif chemokine 19-4	Immune Chemokine	ssa24:41009562-41020152	0.001
Interleukin-6 receptor subunit beta; IL6ST; IL6RB	Immune Cytokine receptor	ssa01:149796152-149819776	0.010
Ornithine decarboxylase	Immune Effector	ssa09:27129400-27134433	0.004
Arginase, type II	Immune Effector	ssa09:48189270-48205393	0.002
lipocalin-like	Immune Eicosanoid	ssa11:78362795-78377849	0.006
CD9-1	Immune IFN-virus response	ssa06:31233349-31244616	0.006
Very large inducible GTPase 1-2	Immune IFN-virus response	ssa06:33802332-33807836	0.001
ATP-dependent RNA helicase DHX58	Immune IFN-virus response	ssa06:37848177-37853413	0.004
Sacsin	Immune IFN-virus response	ssa09:25211291-25239564	0.001
Tyrosine-protein kinase Jak1	Immune IFN-virus response	ssa14:8762239-8786214	0.002
VHSV-induced protein-1	Immune IFN-virus response	ssa14:59522300-59526637	0.006
Interferon-induced protein 44-1	Immune IFN-virus response	ssa16:43549789-43558483	0.010
STAT1a	Immune IFN-virus response	ssa16:64447882-64461741	0.005
Nicotinamide phosphoribosyltransferase-1	Immune IFN-virus response	ssa17:37594963-37613759	0.010
E3 ubiquitin-protein ligase KEG-like	Immune IFN-virus response	ssa18:53454158-53470782	0.002
Plac 8-like	Immune IFN-virus response	ssa18:53541267-53555751	0.008
Interferon-induced very large GTPase 1	Immune IFN-virus response	ssa18:55753718-55764224	0.006
interferon regulatory factor 3	Immune IFN-virus response	ssa19:63391614-63418578	0.007

Aquaporin-1	Immune IFN-virus response	ssa20:7077184-7086819	0.003
proteasome beta 8 subunit E2	Immune IFN-virus response	ssa27:10153895-10157742	0.001
Ubiquitin-like protein-2	Immune IFN-virus response	jcf1000235881_0_0:4869-5879	0.000
Barrier-to-autointegration factor (banf)	Immune IFN-virus response	jcf1000703746_0_0:6683-9930	0.005
Ig light chain	Immune Ig	ssa17:48525511-48527477	0.001
immunoglobulin mu heavy chain	Immune Ig	ssa26:35488111-35489575	0.002
C-type lectin domain family 4 member E-like	Immune Lectin	ssa27:7678810-8014319	0.004
lymphocyte cytosolic protein 2-like	Immune Lymphocyte	ssa04:74125077-74138110	0.009
Myeloid-associated differentiation marker	Immune Lymphocyte	ssa14:34162787-34171481	0.000
SLAM family member 7 precursor	Immune Lymphocyte	ssa17:18383186-18399153	0.001
Platelet-activating factor acetylhydrolase 1b, catalytic subunit 2	Immune Platelet	ssa02:15565305-15574901	0.004
CD9 protein	Immune Platelet	Ssa03:64774491-64787861	0.000
72 kDa type IV collagenase, mmp2	Immune protease	ssa11:29650370-29671526	0.002
Matrix metalloproteinase-9	Immune protease	ssa13:6141900-6149211	0.002
Inactive serine protease PAMR1	Immune protease	ssa16:20603741-20673034	0.000
toll-like receptor 12	Immune receptor	ssa02:3311985-3322330	0.004
CCAAT/enhancer binding protein (C/EBP)_ beta	Immune regulator	ssa13:40482787-40484397	0.008
V-yes-1 Yamaguchi sarcoma viral oncogene homolog 1	Immune regulator	ssa27:23069463-23093234	0.003
Plastin 3	Immune T cell	ssa05:611711-665090	0.007
T cell immune regulator	Immune T cell	ssa09:65568701-65579911	0.008
Interferon-related developmental regulator 1	Immune T cell	ssa15:63358567-63374840	0.005
CD82 antigen, a	Immune T cell	ssa16:1165157-1222830	0.004
Interleukin-4 receptor alpha chain	Immune T cell	Ssa03:62451522-62460089	0.001
Jun B-1	Immune TNF	ssa06:29993781-29995614	0.009
TRAF-type zinc finger domain-containing protein 1	Immune TNF	ssa09:103077297-103081324	0.005
lipopolysaccharide-induced tumor necrosis factor-alpha factor homolog	Immune TNF	ssa12:8352041-8354399	0.002
TNF decoy receptor	Immune TNF	ssa15:68040802-68043031	0.010
tumor necrosis factor receptor superfamily member 5-like	Immune TNF	ssa15:94963567-94979084	0.001

Protein prune homolog	Immune TNF	ssa20:18336763-18388054	0.007
Potassium channel tetramerisation domain containing 10	Immune TNF	Ssa03:48384858-48420925	0.006

***Supplemental table S3. Highlighted immune genes with differential expression in H- versus L-gEBV salmon associated with SNP allele at locus* AX-98325256 (Ssa03 position 904438097) and AX-98325250 (Ssa03 position 90438576).** *Chromosome, start and end map base positions for each gene are shown.*

**Gene**	**Function**	**Position**	**AX_98325250 p_value**	**AX_98325256 p_value**
NACHT, LRR and PYD domains-containing protein 1 (stonustoxin subunit beta-like)	Immune	jcf1000721884_0_0:34279-43741	1.85E-09	4.85E-09
lipopolysaccharide-induced tumor necrosis factor-alpha factor homolog (litaf-homolog)	Immune Cytokine	ssa06:13143954-13160089	1.80E-05	2.29E-05
GLI family zinc finger 3 (gli3)	Immune IFN-virus response	ssa05:71173230-71175948	0.003	0.003
Type-2 ice-structuring protein (isp2)	Immune Lectin	ssa08:20195570-20197752	0.004	0.004
Ladderlectin	Immune Lectin	jcf1000227469_0_0:4537-9463	0.006	0.006
